# Bias‐Tunable Two‐Terminal Organic Photodetector for Intelligent Imaging

**DOI:** 10.1002/advs.75894

**Published:** 2026-06-01

**Authors:** Sangin Hahn, Sanghoon Park, Seunghyup Yoo

**Affiliations:** ^1^ School of Electrical Engineering Korea Advanced Institute of Science and Technology (KAIST) Daejeon Republic of Korea

**Keywords:** in‐sensor computing, organic optoelectronics, reconfigurable photodetector, thin‐film photodetector

## Abstract

Image sensors capable of in‐sensor computing can substantially reduce computational burdens in machine vision systems that require extensive data post‐processing. Reconfigurable photodetectors, which serve as the fundamental building blocks for such sensors by enabling pixel‐level signal modulation, are therefore central to this development. They have been explored using emerging materials or modified field‐effect transistor architectures, but their complex structures and processing limit scalability and resolution. Here we report a reconfigurable photodetector with a simple, vertically stacked two‐terminal structure based on an organic donor/acceptor/donor tri‐layer active, which functions equivalently as dual photodiodes connected with opposite polarity with a resistor inserted between them. The device exhibits 80 dB linear dynamic range and, furthermore, features responsivity that can be tuned in a quasi‐linear fashion from −26 mA/W to 77 mA/W under operation bias from −1 V to 1 V. This continuously variable responsivity can act as weight values in a neural network, as demonstrated with a 4 × 4 photodetector array that performed reconfigured photocurrent map generations and kernel operations, emulating simple image preprocessing filters. Its voltage‐controlled tunability and straightforward fabrication process underscore the potential for monolithic integration with various backplane technologies, paving the way toward compact and efficient intelligent image sensors.

## Graphical abstract

A vertically stacked two‐terminal photodetector with a symmetric donor–acceptor–donor trilayer active exhibits reconfigurable photoresponse with bias‐tunable magnitude and polarity, together with sub‐millisecond response speed and a wide tunable output window. Leveraging scalable process compatibility, a 4 × 4 photodetector array is fabricated and demonstrated for kernel operations, offering a scalable platform for compact intelligent image sensors.

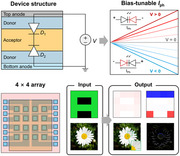

## 1 Introduction

While conventional image sensors have made remarkable progress, there is a growing interest in developing sensing paradigms with more sophisticated controllability. Advanced machine vision technologies are widely investigated for a broad range of applications, particularly for tasks such as industrial, agricultural, and security monitoring that require complex processing of raw image data [1, 2, 3, 4, 5, 6] For efficient processing and faster response speed, the demand for resolutions and refresh rates of input data has increased rapidly, thus resulting in substantial amounts of computational cost and power consumption. In this regard, the incorporation of in‐sensor computing capability into image sensors has recently been proposed as a promising solution. Integrating data processing capabilities directly within the sensor hardware enables the immediate processing of captured data without the need for external processors. This approach addresses the growing challenges of data bottlenecks and energy inefficiency in conventional sensing systems with complex computational post‐processing steps [7, 8, 9, 10]. Therefore, power consumption and overall system response time could be improved, offering crucial advantages for the complex imaging applications described above. Furthermore, it also enables unique functionalities such as on‐chip machine learning and adaptive sensing [11, 12].

As a crucial component for achieving such features, researchers have explored various reconfigurable photodetectors that can modulate the scale and the polarity of photocurrent responsivity, which is in contrast to conventional photodiodes with fixed photocurrent response in the saturattion region [8, 13, 14]. With dynamic adjustment of their response characteristics, these reconfigurable devices can perform low‐level computing at the sensor level. Such features are especially beneficial for hardware‐integrated deep neural networks (DNNs) and convolutional neural networks (CNNs) [7, 8, 9, 10] In such systems, front‐end convolution operations are usually performed for feature extraction and thereby constructing feature maps from input images. During the convolution process, kernels for target features are applied and scanned across the input image. Kernels are composed of weight values, returning a convolved output as an extracted feature value at the specific location. In the reconfigurable photodetector array, this could be performed within the array itself. In this case, each photodetector dynamically adjusts the responsivity as a weight value in the kernel, while the input image is considered as an irradiance map. As a result, the convolved output of the kernel at the specific location could be collected as a summation of photocurrents from each pixel, thus implementing the direct process of input data [14, 15, 16, 17] Furthermore, since kernels with both positive and negative weights have been shown to have better learning efficiency and network performance, it is crucial to realize bidirectional photoresponse within the detector itself [18, 19].

In previous works, field‐effect transistor (FET) based devices have particularly been preferred for implementing reconfigurable photodetectors due to their intrinsic channel tunability through controlling the gate voltage, thus realizing reconfigurable photoresponse. In particular, 2D materials were mainly chosen for achieving ambipolar photoresponse due to their unique electrical characteristics, such as high carrier mobility and the ability to achieve both n‐type and p‐type conduction [20, 21, 22, 23, 24, 25]. Moreover, electrostatically doped devices that can modulate the direction of band bending and current flow were also suggested [26]. However, such devices with complicated structures of three or four‐terminal operation schemes or complex fabrication processes tend to make it complex to integrate them with readout backplanes, thereby limiting the realization of a reconfigurable photodetector array with a line addressing operation scheme. In this respect, various two‐terminal devices have been designed to achieve better integration capabilities. These include UV‐sensitive reconfigurable photodetectors with wide‐gap photoactive materials and devices with programmable short‐circuit photocurrent by poling [27, 28]. Despite these advances, further improvements are required, particularly in extending the spectral response range, realizing scalable fabrication processes, and establishing operation schemes without the need for pre‐programming steps before actual sensing.

Herein, we propose a vertically stacked two‐terminal organic reconfigurable photodetector with a bias‐controllable photocurrent response. To realize controllable bidirectional photoresponse, we adopt an organic tri‐layer structure consisting of donor/acceptor/donor structures, which turns out equivalent to oppositely stacked dual photodiodes with an equivalent resistor inserted between them. While oppositely stacked two photodiodes in configurations of donor/acceptor/intermediate electrode/acceptor/donor have been previously exploited mainly for dual‐mode photodetector application [29, 30, 31, 32, 33, 34], the present work differs in that the proposed devices adopt a simpler, tri‐layer architecture and that they are explored as reconfigurable photodetectors for intelligent imaging. In the proposed scheme, excitons are generated at donor/acceptor planar heterojunctions located at both sides of the middle acceptor layer, and only one kind of carriers are allowed to flow through the device according to the polarity of the bias, enabling a wide range of tunability for responsivity from negative to positive values [35, 36, 37]. Meanwhile, the dark current, which occurs due to charge injections from electrodes, is suppressed by energy barriers offered by interlayers, laying a foundation for a large dynamic range.

An actual device with 1,1‐Bis[(di‐4‐tolylamino)phenyl]cyclohexane (TAPC) donor and C_70_ acceptor is fabricated and has shown dynamically controllable responsivity range from −26 mA/W to 77 mA/W under operation voltage range from −1 to 1 V. Due to its simple structure, the proposed photodetector can be fabricated with low‐cost, scalable fabrication techniques, offering better integration capabilities with various backplanes than conventional in‐sensor computing architectures that require multiple transistors. We have also fabricated the 4 × 4 reconfigurable photodetector array, showing uniform photocurrent under both positive and negative bias conditions. Moreover, reconfigured photocurrent maps of the input pattern were successfully generated. These results and current summation output generation illustrate the potential adaptability of designed photodetectors for the kernel operation within the front‐end edge computing. To validate the in‐sensor computing capability, we computationally constructed and successfully trained a simple neural network for image classification based on the actual photoresponse characteristics of the photodetector. These results confirm the feasibility of the proposed devices for intelligent image sensors with in‐sensor computing capability.

## 2 Results and Discussion

### 2.1 Design Concept for Thin‐Film Reconfigurable Organic Photodetectors

**FIGURE 1:**
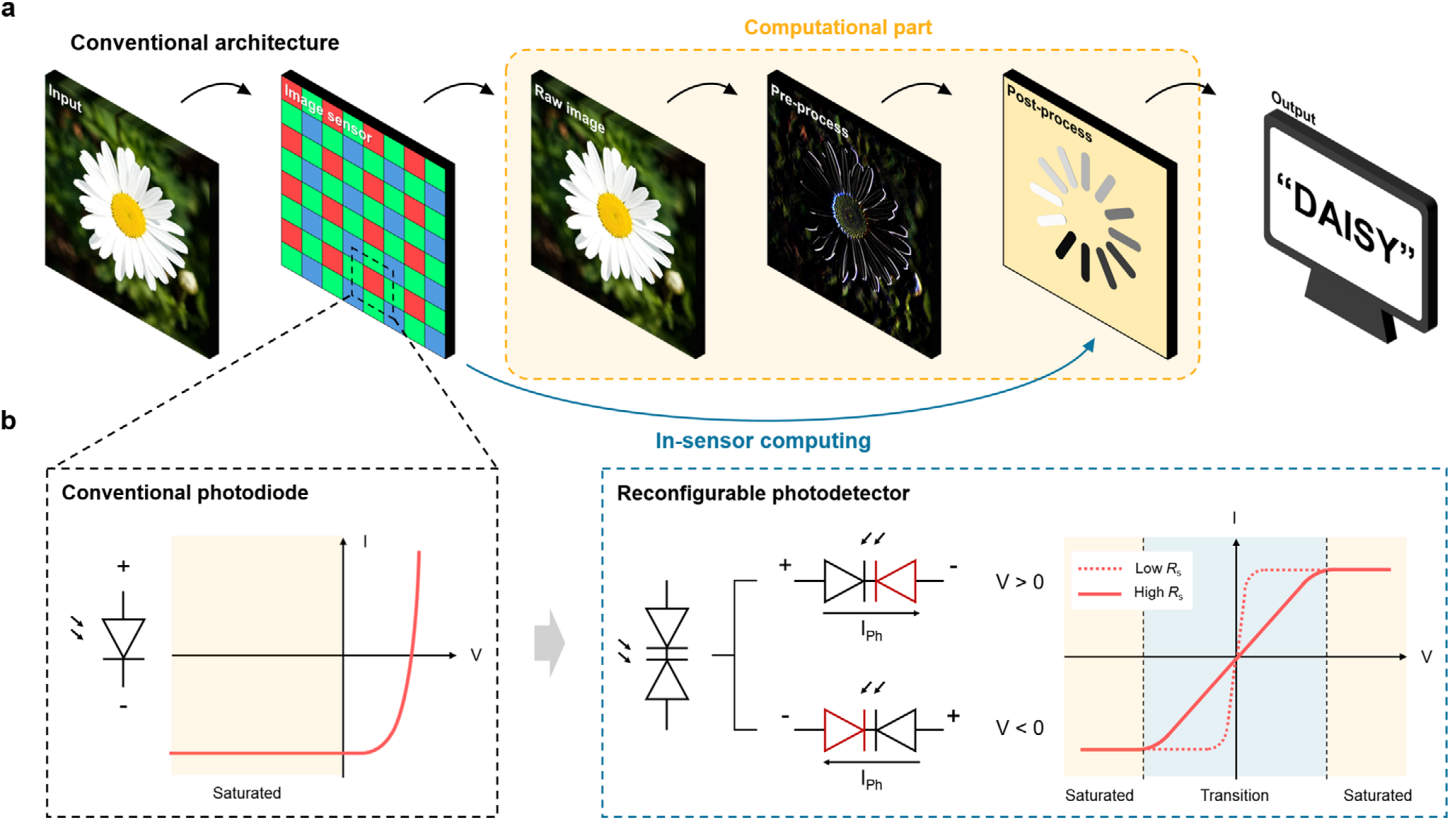
Schematic illustration of reconfigurable photodetectors for in‐sensor computing beyond conventional imaging. (a). Overview of image capturing and processing. In conventional systems, the input optical images are converted into digital raw data by the image sensor and subsequently processed through external computational stages. In contrast, in‐sensor computing with reconfigurable photodetectors integrates image preprocessing within the photodetector array in a controllable manner. (b). Conventional photodiodes in pixels exhibit a fixed photocurrent response in reverse bias conditions(left), whereas the proposed reconfigurable photodetector can dynamically adjust photocurrent response depending on the operation bias(right).

With their controllable responsivity, reconfigurable photodetectors can serve as core building blocks for image sensors that move beyond conventional image capturing to include built‐in computing capabilities such as feature extraction for object identification (Figure 1a). To realize a device capable of exhibiting photocurrent responses with controllable magnitude and polarity, one may begin with the concept of symmetrically stacked photodiodes (PDs) with opposite polarities, thereby enabling bi‐directional photocurrent acquisition, as depicted in Figure 1b. When an operating bias is applied, one PD operates in the reverse bias, while the other works in the forward bias. The reverse‐biased PD generates photocurrent under illumination and exhibits reverse saturation current in the dark, whereas the forward‐biased PD allows current to flow through the whole device while maintaining current continuity between the two PDs. Owing to the identical back‐to‐back configuration, the functional roles of the PDs can be interchanged by simply reversing the bias polarity. This leads to symmetric bidirectional photocurrent responsivity through bias‐selective operation, as illustrated in the schematic current (*I*)‐voltage (*V*) characteristics shown on the right side of Figure 1b. When there is non‐zero equivalent series resistance between the stacked PDs, in particular, the ‘transition’ region becomes wider than the zero‐resistance case, making it convenient to control the responsivity in a linear fashion via applied bias, as compared in Figure 1b.

**FIGURE 2:**
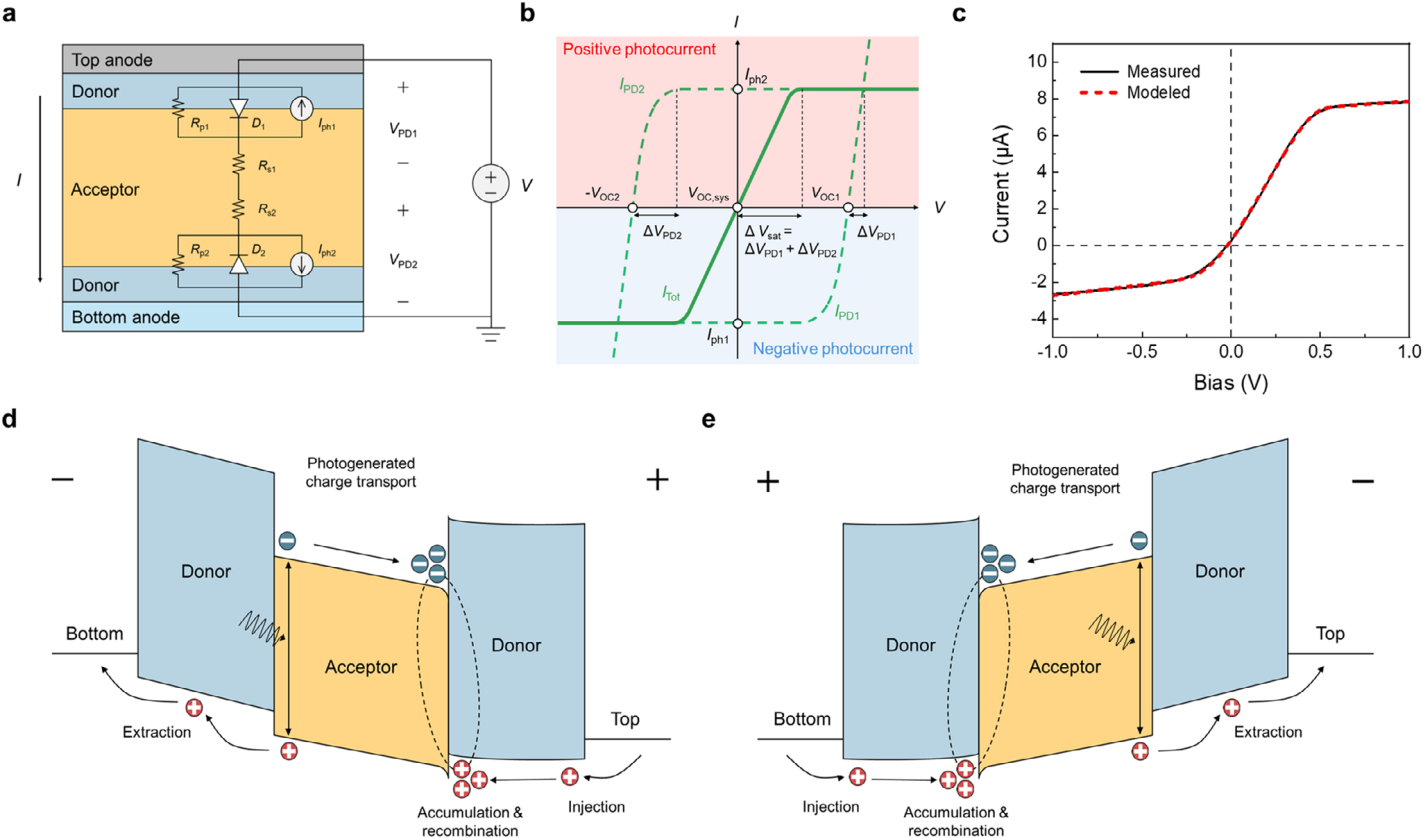
Design concept of the suggested reconfigurable photodetector. (a). Bias‐tunable thin‐film reconfigurable photodetector with tri‐layer active structure with an equivalent circuit. (b). Photocurrent response characteristics of the suggested photodetector with top and bottom photodiodes. (c). Measured photocurrent response of the actual device and computationally fitted curve with the equivalent circuit model. (d). Excited charge generation and transportation scheme under forward bias, also suppression of the charge injection from electrodes is depicted. Positive bias is applied to the left side of the device. (e). Behavior of the photodetector under reverse bias.

Since organic photodiodes are designed with donor (D) /acceptor (A) heterojunction interfaces as rectifying junctions, the suggested series‐connected photodiodes in the back‐to‐back configuration could be realized in a form of anode_1_/ D/ A/ cathode/ A/ D/ anode_2_ or in a form of cathode_1_/ A/ D/ anode/ D/ A/ cathode_2_. For the sake of simplicity in both fabrication and device optimization, we propose to simplify the device structure by omitting the intermediate common electrode and merging the middle A or D layers (Figure 2a). In the D/ A/ D configuration, for instance, D/A and A/D planar heterojunctions are formed on both sides of the middle A layer, making the device virtually equivalent to two vertically stacked PDs with opposite polarities.

### 2.2 Device Operation Scheme

When the bias *V* is applied, it is split to the top and the bottom photodiodes (*V* = *V*_PD1_+*V*_PD2_). Under the open‐circuit voltage condition (*I* = 0), the top and bottom photodiodes are also in the open‐circuit voltage without current. (*I* = *I*_PD1_ = *I*_PD2_ = 0, *V*_PD1_ = *V*_OC1_, *V*_PD2_ = ‐*V*_OC2_) In the ideal symmetric configuration, the open‐circuit voltage of the photodetector system as a whole (*V*_OC,sys_) should be zero as shown in Figure 2b, assuming the same level of photocurrent is generated from each of the PDs. From this point, when the operation bias is shifted further forward (i.e. *V* > *V*_OC,sys_), the bias across the top photodiode would be shifted further forward and the bottom PD would be shifted further backward. The current level of the top PD would be exponentially increased, while the bottom PD would be shifted toward the saturation region, therefore showing bias‐tunable photocurrent response before the saturation point. When the device reaches the saturation level (Δ*V*_sat_), the current level of the bottom PD is saturated to *I*_ph2_, but that of the top PD cannot be further increased due to the current that matches the saturation current of the bottom PD. When the operation bias is further applied and higher than the onset voltage of the saturation level, additional voltages are applied to the bottom PD since it shows a constant current level after entering the saturation region, while the bias applied to the top photodiode is fixed. Consequently, the designed photodetector exhibits, under illumination, *I‐V* characteristics similar to the error function, including a transition region with a bias‐dependent tunability therefore offers the opportunity to operate the device as a reconfigurable photodetector. In the dark condition, both *I*_ph1_ and *I*_ph2_ are zero, thereby showing the identical but far suppressed characteristics in which the saturation region is limited by the reverse saturation current of each PD.

In real cases, however, *I‐V* characteristics of organic PDs (OPDs) are often far from the ideal diode behavior given by Shockley equation and could be modelled with an equivalent circuit containing both series and shunt resistances (*R*_s_ and *R*_p_), as depicted in Figure 2a. Practically, the effective series resistance reflects the cumulative transport or contact bottleneck of the device, arising from the finite conductivity of the organic transport layers and the contact resistance at the electrodes, etc. The shunt resistance, on the other hand, represents any process that can add effective current flow exhibiting linear dependence on bias to a good approximation (e.g. leakage current) [38, 39, 40, 41]. In this case, one can set up the following set of nonlinear simultaneous equations for the photodetector system:

(1) $$V=V_{\mathrm{PD}1}+V_{\mathrm{PD}2}$$

(2) $$I=I_{\mathrm{PD}1}=I_{\mathrm{PD}2}$$

(3) $$\begin{array}{ccc} & = & \frac{V_{\mathrm{PD}1}-IR_{s1}}{R_{p1}}+I_{\mathrm{ph}1}-I_{D1}=\frac{V_{\mathrm{PD}1}-IR_{s1}}{R_{p1}}-I_{\mathrm{ph}1}\\ & & \;+\;I_{s1}\left[\exp\left(\frac{q\left(V_{\mathrm{PD}1}-IR_{s1}\right)}{n_{1}kT}\right)-1\right] \end{array}$$

(4) $$\begin{array}{ccc} & = & \frac{V_{\mathrm{PD}2}-IR_{s2}}{R_{p2}}+I_{\mathrm{ph}2}-I_{D2}=\frac{V_{\mathrm{PD}2}-IR_{s2}}{R_{p2}}\\ & & \;+\;I_{\mathrm{ph}2}-I_{s2}\left[\exp\left(-\frac{q\left(V_{\mathrm{PD}2}-IR_{s2}\right)}{n_{2}kT}\right)-1\right] \end{array}$$

where *R*_s1_ and *R*_s2_ are the series resistances, *R*_p1_ and *R*_p2_ are the shunt resistances, *I*_ph1_ and *I*_ph2_ are photocurrents, *I*_s1_ and *I*_s2_ are saturation currents, *n*_1_ and *n*_2_ are ideality factors of the top and the bottom PDs.

The system of nonlinear implicit equations was solved numerically, and the fitting parameters could be calculated by using a nonlinear least‐squared error algorithm. As the reverse characteristics of OPDs are often influenced by low *R*_p_, the current in the saturation region is not likely to be flat but tends to have a slight slope, while the slope within the transition region and its width are influenced by non‐zero *R*_s_ as discussed earlier. Furthermore, a difference in the photocurrent may yield non‐zero *V*_OC,sys_. Such departure from the ideal, symmetrical characteristics are indeed observed in the present system as shown in Figure 2c, and the experimental characteristics are well fitted to the equivalent circuit model discussed above (Figure S1 and Table S1). Notably, *R_s_* values of both photodiodes were relatively high compared to those of the previously reported thin‐film photodiodes [38, 39, 40]. We have addressed that in the vertically stacked D/A/D architecture, carriers must traverse two rectifying interfaces and the intermediate organic layer that leads to charge accumulation and transport limitation. Particularly, photogenerated electrons on reverse‐biased junction are accumulated on the forward‐biased D/A heterojunction and recombined, instead of directly collected to the electrode through forward bias region and thus increasing the effective collection resistance as illustrated in Figure 2d. Therefore, the unusual level of 𝑅_𝑠_ extracted from the fitting should be understood as a lumped parameter reflecting the various sources of internal transport bottleneck present in the proposed photodetector system. In this way, the designed device exhibits a lower slope within the transition region than that of the single photodiode in the forward bias region, ensuring a wide bias window for controlling the responsivity.

### 2.3 Energy Band Alignment and Charge Transport Mechanism

To provide a better understanding of the modeled *I*‐*V* characteristics, the detailed analysis of the device was further investigated for charge transport and energy level alignment. Figure 2d,e illustrate the behavior of the proposed device under illumination in terms of charge generation and transport with energy band diagrams. When the forward bias is applied, the top OPD is in the forward bias, whereas the bottom OPD is in the reverse bias. These operation conditions affect both D/A interfaces, resulting in the band bending depicted in Figure 2d. The bottom donor/acceptor shows band bending like that of OPDs in the reverse bias, and the top donor layer is almost flat.

After excitons are separated in the D/A interfaces to holes and electrons, the bottom donor layer transports photogenerated holes, and the top donor layer enables the transport of injected holes from the top electrode to the top D/A interface. On the other hand, electrons are transported through the acceptor layer to the top D/A interface and accumulated therein because of the potential barrier. At the donor side of the same interface, injected holes from the top electrode are accumulated, thereby getting recombined with electrons and inducing a slight level of local band bending. Consequently, the device generates photocurrent responses predominantly as a hole‐only device based on the collection of photogenerated holes on the bottom side and the same level of hole injection on the top side, while electrons are recombined within the interface.

Such a tri‐layer active configuration not only efficiently generates photocurrent signals but also tends to reduce the dark current originating from charge injections from electrodes. The potential barriers formed by the donor layers suppresses electron injection from the bottom electrode, while the potential barriers created by the acceptor material blocks the holes injected from the right electrode from moving through the device. Furthermore, due to the near‐symmetric design of the device, the direction of the photocurrent can be easily controlled by simply changing the polarity of the applied bias (Figure 2e).

### 2.4 Experimental Realization and Characterization

**FIGURE 3:**
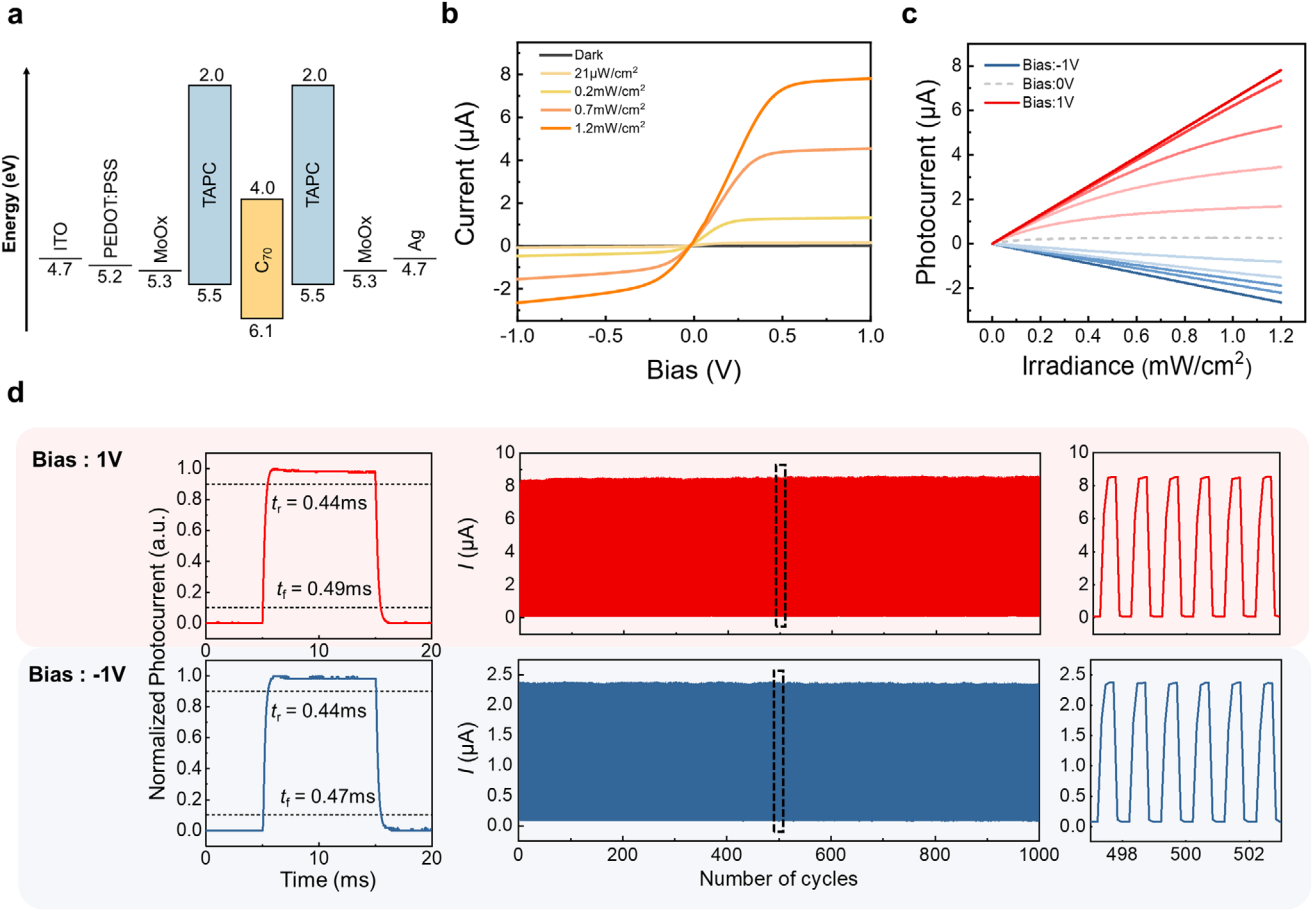
Fabricated reconfigurable thin‐film photodetector with an organic tri‐layer structure. (a). Schematic energy diagram of the layers involved in the photodetector based on 1,1‐Bis[(di‐4‐tolylamino)phenyl]cyclohexane (TAPC) as a donor and C_70_ as an acceptor. (b). *I*‐*V* curves under different irradiance conditions. The green LED (peak wavelength of 535 nm) was utilized as a light source, and the active area of the device is 0.085 cm^2^. (c). Linearity of the photocurrent with respect to the operation bias conditions of zero, ±0.1, ±0.2 ±0.3, ±0.5 and ±1 V. d. Transient photocurrent response characteristics (left) and photocurrent stability during 1,000 cycles (right), under +1 V and −1 V of operation bias.

An actual organic reconfigurable photodetector was designed and fabricated as a two‐terminal bottom‐illumination device in the configuration of glass | ITO 150 nm | PEDOT:PSS (Clevios P VP Al 4083) 40 nm | MoO_x_ 10 nm | TAPC 60 nm | C_70_ 87 nm | TAPC 60 nm | MoO_x_ 10 nm | Ag 100 nm. We selected TAPC and C_70_ as the donor and acceptor layers, respectively, since they constitute one of the well‐established organic donor/acceptor combinations and provide a well‐understood energy‐level framework for constructing vertically stacked D/A/D devices. In addition, the wide band gap of TAPC and the strong photoactive/acceptor characteristics of C_70_ are favorable for realizing carrier‐selective transport and interfacial charge separation in the present architecture. The energy levels of each material is presented in Figure 3a [42, 43, 44, 45]. To experimentally verify the energy level alignment at the actual interfaces, UV photoemission spectroscopy (UPS) was performed on the deposited MoO_x_, TAPC, and C_70_ films to determine the work functions and valence‐band edge positions, while the optical bandgaps were estimated from the corresponding Tauc plots (Figures S2 and S3). Based on these measured parameters, the energy‐level diagram in Figure 3a was constructed, and the resulting values are summarized in Table S2 with the detailed band diagram after junction formation provided in Figure S4 [46, 47]. To ensure long‐term stability, the devices were further encapsulated with an Al_2_O_3_ layer of 30 nm thickness via atomic layer deposition (ALD), based on previous works reported that barrier performance typically saturates at this scale. The efficacy of the encapsulation was verified by monitoring the photocurrent response of both encapsulated and bare devices under ambient conditions. While the bare devices exhibited significant degradation due to the transmission of moisture and oxygen into the organic active layer, the encapsulated devices maintained over 96% of their initial photocurrent throughout the 10‐day test period (Figure S5) [48, 49, 50].

In the present device configuration, the middle C_70_ layer serves not only as the main photoactive absorber but also as the central layer that defines two identical C_70_/TAPC heterojunctions for charge generation and separation. Under illumination, excitons are generated predominantly in C_70_ and dissociate at both interfaces, while the wide‐gap TAPC donor layers hardly absorb visible light. After carrier generation, the TAPC layers selectively transport holes to the targeted electrodes and suppress unwanted electron injection from the electrodes by offering interfacial barriers at the C_70_/TAPC heterojunctions. Notably, these interfacial barriers also induce charge accumulation and facilitate recombination at the forward‐biased heterojunction, thereby enabling selective collection of photogenerated holes from the reverse‐biased heterojunction and a bidirectional photocurrent response with bias‐switchable polarity. Notably, MoO_x_ layers also play a role as an anodic buffer layer by providing optimal energy level alignment with adjacent TAPC layers thus energy levels of each layer are aligned with its work function [51, 52]. (Figure S4) PEDOT:PSS was introduced between ITO and MoO_x_ to further facilitate efficient hole injection and extraction to the bottom side. Consequently, the device can successfully generate a photocurrent signal in this hole‐only operation mode, and its direction can also be controlled simply by changing the polarity of the applied bias. It is also noteworthy that the proposed operation mechanism can be further validated by the TCAD simulations (ATLAS, Silvaco), which further explain the internal field distribution and carrier accumulation near the C_70_/TAPC interfaces under opposite bias polarities, consistent with the experimentally observed asymmetric transport behavior (Figure S6 and Table S3).

Since the device operates as the photodetector by generating the current signal under illumination, detailed measurements related to the photocurrent (*I*_ph_), defined as *I*_ph_ = *I*_light—_ *I*_dark_, were performed and are illustrated in Figure 3b–d. Here, *I*_light_ denotes the current in the illuminated condition, while *I*_dark_ denotes the current in the dark condition. From the current‐voltage (*I*‐*V*) characteristics of the fabricated device with the bias voltage swept from −1.0 to 1.0 V with a step of 0.01 V (Figure 3b), we note that the fabricated device has shown a low dark current density below 1 µA/cm^2^ (Figure S7) and the measured noise spectral density was 6.48 × 10^−12^ AHz^−1/2^ at 100 Hz (Figure S8) in the room temperature, thus yielding the specific detectivity of 3.46 × 10^9^ Jones, while successfully generating a bidirectional photocurrent response with bias‐controllable magnitude and polarity [40, 53]. The photocurrent gradually increased, across the range of the bias applied in this work, when the irradiance of the incident light (*P*_inc_) varied from 21 µW/cm^2^ to 1.2 mW/cm^2^ (Figure 3b). For a fixed voltage, the photocurrent exhibited linear response under *P*_inc_ from 133.3 nW/cm^2^ to 1.2 mW/cm^2^, ensuring a linear dynamic range (LDR) of 80 dB or higher at 1 V bias (R^2^> 0.99, Figure 3c). The responsivity was calculated from the measurement using the following equation [54]:

(5) $$R\;\left[A/W\right]=\frac{I_{\mathrm{ph}}}{P_{\mathrm{inc}}\times A}=\frac{I_{\mathrm{light}}-I_{\mathrm{dark}}}{P_{\mathrm{inc}}\times A}$$

where *A* is the light‐absorbing area, and it was found to range from—26 mA/W to 77 mA/W under an operating bias of −1 V to +1 V, confirming the device successfully operates as a reconfigurable photodetector. Notably, the level of the obtained responsivity values are much lower than those of a C_70_/TAPC planar heterojunction photodiode (0.17 A/W at 535 nm, Figure S9). The lower responsivity of the present tri‐layer device reflects its intentionally engineered carrier transport pathway, in which interfacial potential barriers induce carrier accumulation and recombination rather than direct extraction, thereby enabling bias‐controlled bidirectional tunability at the expense of absolute sensitivity. In addition, although the proposed D/A/D structure appears geometrically symmetric in Figure 2a, its optical and electrical responses are not strictly symmetric partly because the distribution of the internal optical field is not symmetric between the top and bottom OPDs and is wavelength‐dependent. Accordingly, the difference between the two polarities should be understood as a consequence of wavelength‐dependent field localization and polarity‐dependent carrier collection in the stacked structure. To quantitatively evaluate this effect, we measured the refractive index and extinction coefficient spectra of each layer by ellipsometry and calculated the optical field distribution using the transfer matrix method. (Figure S10) This analysis shows that the upper junction near the Ag side is more strongly involved under negative bias, whereas the lower junction near the ITO side contributes more significantly under positive bias. As a result, the device preferentially collects shorter wavelength photoexcitation under ‐1 V and longer wavelength photoexcitation under +1 V, leading to the observed asymmetry in responsivity. (Figure S11) To support this notion, we further measured *I‐V* characteristics under 467 nm LED illumination. Under the illumination of this blue light, *I‐V* curve has shown improved symmetrcity, responsivity ranging from −40 mA/W to 63 mA/W under operation −1 to 1 V bias range. (Figure S12) In addition to this optical‐field asymmetry, the fitting parameters in Table S1 also reflect different electrical transport conditions in the upper and lower junctions, including asymmetric series resistances which represent distinct carrier collection in each electrode (*R*_s1_ *A* = 2.29 × 10^3^ Ωcm^2^, *R*_s2_ *A* = 1.38 × 10^3^ Ωcm^2^). Therefore, the large difference between −1 and 1 V responsivity values should be understood as the combined consequence of asymmetric field distribution and polarity‐dependent carrier collection in the stacked structure, rather than a contradiction of the nominally symmetric D/A/D geometry.

Figure 3d shows the transient response characteristics to a pulsed laser diode with peak wavelength of 405 nm and pulse width of 10 ms. Under +1 V and −1 V operating bias conditions, the device has shown rise times (*t*_r_; time for photocurrent increases from 10% to 90% of the maximum value) of both 0.44 ms, respectively, while fall times (*t*_f_; time for photocurrent decreases from 90% to 10% of the maximum value) were 0.49 and 0.47 ms. Although the present organic device already operates in the sub‐millisecond regime, its response speed can still be further improved through optimization of the active materials and interfaces. Because the proposed architecture is based on a general thin‐film semiconductor stack, it may be transferable to other material systems with faster carrier transport, thereby enabling broader application spaces, including more speed‐sensitive image‐recognition tasks. The detailed comparison of the unit‐device and array response speeds, together with representative literature values, is provided in Table S4 [12, 21, 26, 27, 55]. Furthermore, the operational stability of the device was measured by undergoing 1,000 cycles of light switching with a frequency of 1 Hz and *P*_inc_ of 1.2 mW/cm^2^. During the cyclic test, the device was operatedunder +1V or ‐1V of constant bias conditions. As a result, the device maintained the constant current levels throughout the whole cycles without significant degradation or hysteresis. After this test, the photodetector exhibited only 2.5% and 0.03% change in *I*_ph_ under +1 V and −1 V bias conditions, respectively. The *I*‐*V* characteristics measured before and after the whole test also confirm the negligible variations of the device (Figure S13). Consequently, the minimal change during the cyclic test in both operating polarities indicates that the proposed photodetector is favorable to long‐term photodetection as well as a repetitive kernel operation for in‐sensor computing.

### 2.5 Photodetector Array Fabrication and Characterization

**FIGURE 4:**
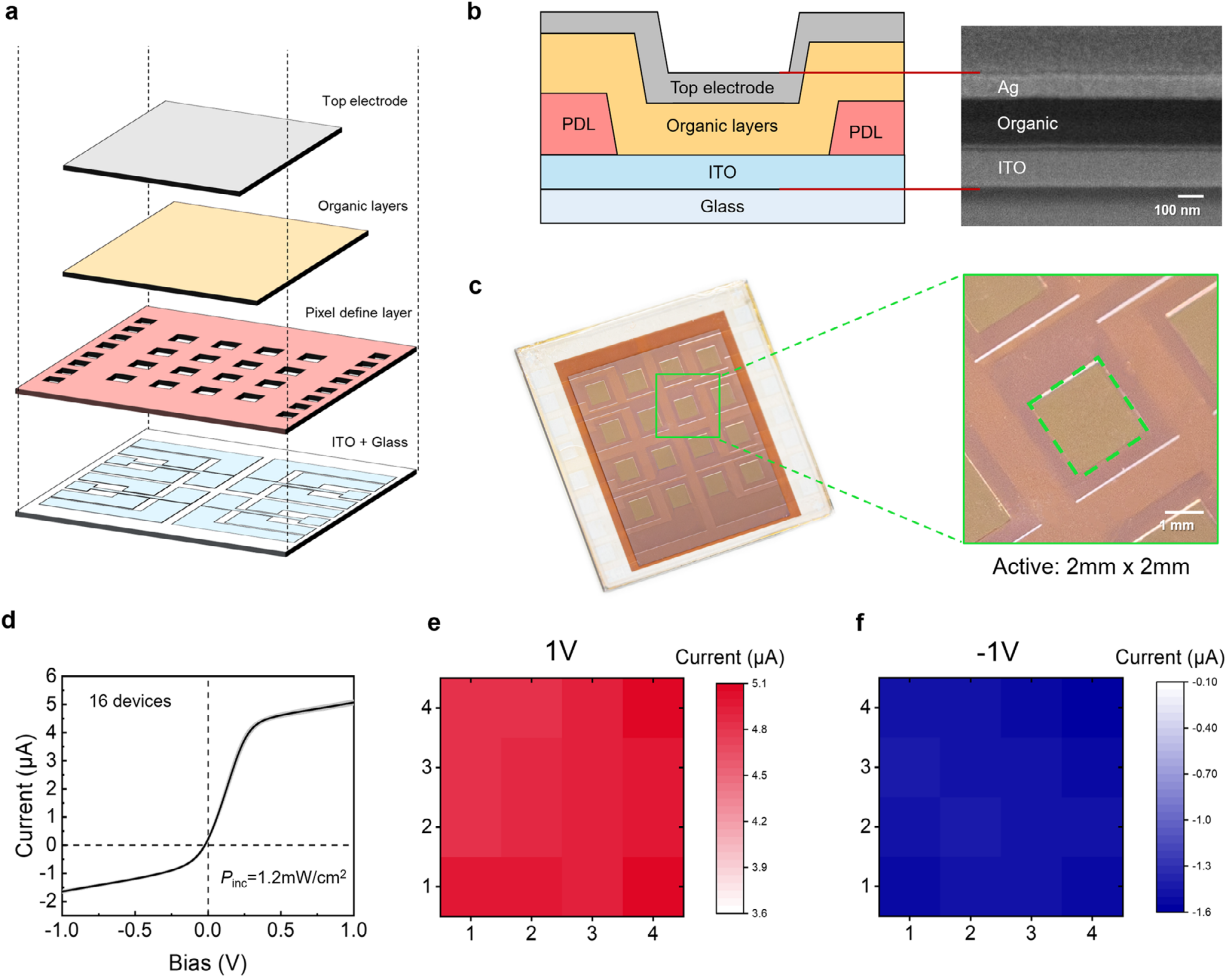
4 × 4 reconfigurable photodetector array on a glass substrate. (a). Schematic illustration of the device structure. (b). Cross‐sectional structure of a single pixel (left) with the corresponding SEM image (right). ‘PDL’ refers to a pixel definition layer. (c). Actual image of the fabricated 4 × 4 photodetector array sample (left) with an active pixel area of 2 mm × 2 mm (right). (d). Average *I*‐*V* characteristics of 16 devices under illumination from a green LED with the peak wavelength of 535 nm. (*P*_inc_ = 1.2 mW/cm^2^), The black line indicates the mean value and the gray region represents the standard deviation, which is so small that the gray region around the solid line is not so visible. e‐f. Current mapping of the array under 1 V (e) and −1 V (f) of operation bias conditions.

As an array‐level demonstration, we fabricated a 4 × 4 reconfigurable photodetector array consisting of 16 individual photodetectors, each having an active area of 2 mm × 2 mm, on a 1‐inch glass substrate. With a pixel‐to‐pixel pitch of 4 mm, the dimension of the total active area of the whole array was 14 mm × 14 mm, which is comparable to the size of conventional image sensors used in modern smartphones. Its vertically stacked structure enabled each pixel to share a top Ag electrode as a common ground. The overall device structure is illustrated in Figure 4a, while the detailed cross‐sectional configuration of a single pixel, including ITO, organic layers, a pixel definition layer (PDL), and the top Ag electrode, is presented in Figure 4b with a corresponding SEM image. The actual image of the fabricated array is provided in Figure 4c. Importantly, the 4 mm^2^ pixel area in the present array is defined by the contact pad opening, which overlaps with the remaining device stack fully covering the active area. This active area determination therefore implies that such thin‐film optoelectronics could be further integrated on a pre‐defined switching backplane without direct patterning of the active layers, which is favorable for high‐resolution array implementation and following pixel miniaturization. To support this, we have compiled representative examples of previously reported backplane‐integrated thin‐film optoelectronics including image sensors and micron‐scale displays in Table S5, with an additional discussion regarding the current stage and further challenges [56, 57, 58, 59, 60, 61, 62, 63]. Additional details about the fabrication process and the layer geometry design are also depicted in Figures S14–S16.

To confirm the uniformity, *I*‐*V* curves of all the pixels were obtained both in the dark condition and under illumination of *P*_inc_ = 1.2 mW/cm^2^ on the sensing plane. The averaged *I*‐*V* characteristics of the 16 devices are shown in Figure 4d, where the black line represents the mean current and the shaded gray region indicates the standard deviation across the array, clearly implying the pixel‐to‐pixel reproducibility of the photocurrent response. In addition, spatial photocurrent maps of the array under +1 V and −1 V operating bias conditions are depicted in Figure 4e,f. These photocurrent maps clearly show that the photocurrent distribution across the active area is highly uniform. The uniformity of the array is represented by the coefficient of variation (CV), which is defined as the ratio of the standard deviation to the mean [64]. The fabricated array exhibited high uniformity with the CV of 1.4% and 1.9% under +1 V and −1 V, respectively. In addition, the pixel within the array has shown a similar level of response speed to the unit device. Measured rise times under 1 V and ‐1 V were both 0.45 ms, while fall times were 0.47 ms and 0.48 ms (Figure S17).

### 2.6 Kernel Operation of Fabricated Photodetector Array

**FIGURE 5:**
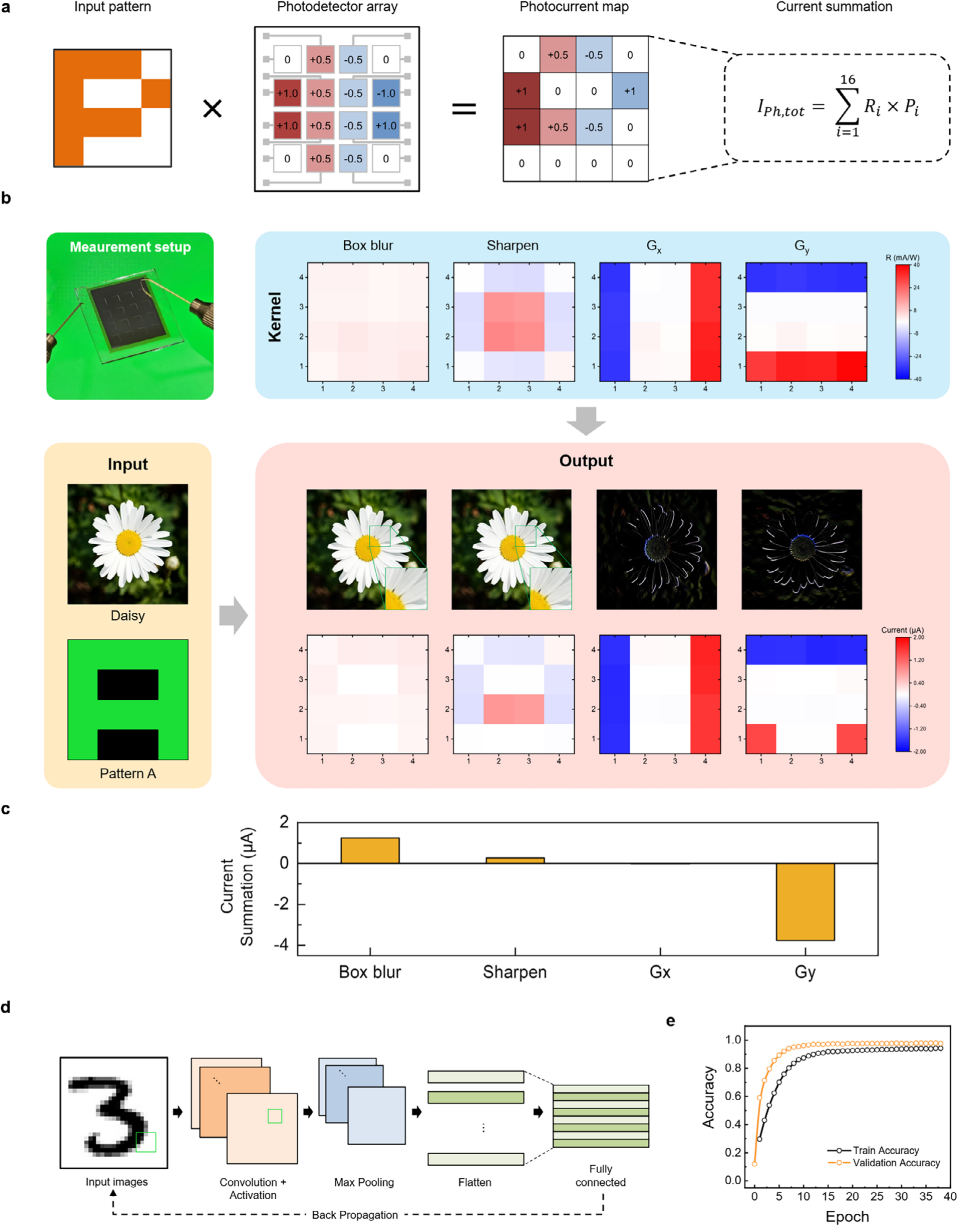
Reconfigured photocurrent map generation. (a). Overview of kernel operation and reconfigured image generation using the fabricated photodetector array (b). Responsivity maps of the photodetector array (top right), functioning as convolution kernels for image preprocessing, were applied to the input pattern (letter “A”) to generate the reconfigured output images shown in the bottom row. For intuitive comparison, the top row illustrates how the kernels would process the daisy image. (c). Summed current values under different kernel operations. (d). The structure of the constructed MNIST handwritten number (0–9) classification, including convolution and pooling steps. e. Training and validation accuracy curves of the constructed neural network.

The reconfigured photocurrent map (image) generation and kernel operation via current summation were performed to validate the in‐sensor computing capability of the fabricated photodetector array. As previously described in Introduction, the responsivity values function as weights in kernels in an analogue manner. Figure 5a provides a brief overview of reconfigurable image generation and the convolutional kernel operation for the array. From projecting the P‐shape 4 × 4 input pattern onto the photodetector array, a designated responsivity map corresponding to a specific kernel operation is applied by dynamically adjusting the operation bias of each pixel, and the resulting photocurrent map is generated. The convolved output is then calculated by summing up the photocurrent signals within the array. In image processing, this operation is performed on each area of the original input image to generate a processed image by a sliding‐window method.

With the fabricated photodetector array, actual kernel operations were performed as described in Figure 5b. Based on average *I*‐*V* characteristics obtained in Figure 4d, appropriate bias voltages for target responsivity values were determined and applied to each pixel, thereby generating reconfigured 4 × 4 images. Well‐known kernels for image preprocessing, such as box blur, sharpen, and horizontal/vertical Sobel filters (G_x_, G_y_) were realized as responsivity maps in the photodetector array [65, 66]. As a result, reconfigured images were successfully generated from each kernel operation based on input pattern ‘A’, and the convolved output signals in Figure 5c were subsequently extracted by summing the current values within the array. From the input pattern, box blur and sharpen operations yielded positive values, while the G_x_ operation produced a value nearly zero, representing that there is no horizontal edge, and the G_y_ operation generated a negative value, indicating the presence of a vertical edge. Those current summation values reflect the intended characteristics of each kernel. (For more intuitive understanding, see also the upper row in Figure 5b that illustrates how these kernels would process the image of a daisy flower.)

These results suggest that the designed reconfigurable photodetector array can effectively modulate its output photocurrent maps and therefore perform low‐level processing through direct convolution implemented as a photocurrent summation. Moreover, the controllable bidirectional photoresponse of the photodetector allows for diverse and efficient edge computing, illustrating its strong potential for realizing image sensors with edge computing features.

### 2.7 Simulated in‐Sensor Computing Capability With the Convolutional Neural Network

Based on the measured reconfigurable photocurrent characteristics and kernel operation results, we constructed a simple convolutional neural network (CNN) for image classification to emphasize the future in‐sensor computing potential of the suggested reconfigurable photodetector array, as illustrated in Figure 5d. The set of MNIST handwritten digit (0∼9) images with 28 × 28 resolution was used for training. Representing the fabricated photodetector array, a set of 4 × 4 kernels was used for the convolution process with the input image. The range of weight values within kernels was controlled to match the representing photocurrent responsivity of the reconfigurable photodetectors (from ‐26 mA/W to 77 mA/W). During the training, weight values were updated through back‐propagation, therefore simulating the responsivity map of the photodetector array repeatedly updated after each epoch (Figure S18). The CNN architecture consists of a convolutional layer with six filters and rectified linear unit (ReLU) activation, max pooling with a 2 × 2 window size, and a fully connected layer with softmax activation for classification. To prevent overfitting, dropout with a 0.1 rate was applied after the pooling layer.

As shown in Figure 5e, the validation accuracy of the constructed CNN rapidly increased, reaching over 90% before 10 epochs from an initial accuracy of 12%. The fast convergence despite the constrained weight range demonstrates that the bidirectional responsivity range in this work is sufficient for effective feature extraction in array‐level neural network operations. Furthermore, to evaluate the robustness of the proposed in‐sensor computing scheme against device non‐idealities, we further performed hardware‐aware CNN simulations incorporating the experimentally observed 10% of pixel‐to‐pixel nonuniformity and 1% of random responsivity fluctuations (Figure S19). The results show that, despite these non‐ideal factors, the learning curves remain close to those of the ideal case, with no significant degradation in convergence behavior or final performance. These results validate that the suggested reconfigurable photodetector could be utilized for realizing a programmable image sensor with in‐sensor computing capability, potentially reducing both the computational load and power consumption compared to conventional digital processing approaches.

## 3 Conclusion

In this work, we demonstrated a thin‐film organic reconfigurable photodetector featuring a simplified vertically stacked, two‐terminal structure with a symmetric donor/acceptor/donor tri‐layer active region. Inspired by the concept of oppositely stacked photodiodes, the device architecture utilizes organic active materials to realize bidirectional photocurrent generation with bias‐controlled magnitude and polarity. We further performed numerical analysis and charge transport modeling based on the energy level diagram to provide a detailed understanding of how the proposed device exhibits such behavior when operated in the transition region. The fabricated devices exhibited a tunable responsivity ranging from −26 mA/W to 77 mA/W under biases between −1 V and +1 V, dynamically adjustable with sub‐millisecond‐scale transient response speed. Moreover, the presented device structure indicates potential spectral tunability through substitution of the active layer materials, potentially extending to other thin‐film optoelectronic materials such as colloidal quantum dots or perovskites. Building on the vertically stacked design and scalable fabrication process, a 4 × 4 array‐level demonstration including kernel operation was conducted. The array exhibited uniform photocurrent distribution and successfully generated preprocessed photocurrent maps by controlling pixel biases according to predefined responsivity maps. These results highlight the potential of the proposed photodetector as a foundational element for intelligent image sensors with in‐sensor computing functionality. To achieve this, further integration of the proposed device with suitable switching backplanes and readout circuits, together with optimal device design frameworks will be required.

## 4 Methods

### 4.1 Fabrication of Reconfigurable Photodetectors

The reconfigurable photodetector was fabricated on a half‐coated 150 nm ITO glass substrate. PEDOT:PSS was spin‐coated on the substrate. Next, a 10 nm‐thick MoO_x_ layer, a 60 nm‐thick TAPC layer, a 87 nm‐thick C_70_ layer, a 60 nm‐thick TAPC layer, and 100 nm‐thick Ag layers were deposited by vacuum thermal evaporation in a vacuum chamber (HS‐1100, Digital Optics & Vacuum) under a vacuum below 1×10^−6^ Torr.

To fabricate the photodetector array, photolithography was processed for patterning bottom electrodes and PDL. A positive photoresist (AZ GXR‐601) was spin‐coated on a 150 nm ITO coated glass substrate and soft baked at 90°C for 3 min. Then, the sample was exposed to UV wavelength of 365 nm with an energy of 22 mJ under the mask for patterning in the mask aligner (MA‐6). The sample was post‐exposure baked at 110°C for 3 min. The sample was developed by a developer (AZ 300 MIF) for 35 sec and rinsed with DI water. The ITO region not covered by the photoresist was etched in an ITO etchant (MA‐S02) for 16 min. After etching, the photoresist was stripped with acetone. To fabricate the PDL on the ITO electrodes, similar patterning process was done. Next, PEDOT:PSS was spin‐coated on the sample, and the other layers were deposited by vacuum thermal evaporation under a vacuum below 1×10^−6^ Torr.

### 4.2 Optoelectronic Measurements of Reconfigurable Photodetectors

The measurements of reconfigurable photodetectors were performed in a nitrogen‐filled glove box under controlled dark conditions using a semiconductor parameter analyzer (HP 4155A, Agilent, Inc). To measure the optoelectronic performance, the devices were illuminated from the bottom side of the substrate by a collimated beam of light from a green LED with a peak wavelength of 535 nm.

## Author Contributions

**Sanghoon Park**: conceptualization, investigation, writing – original draft, methodology, validation, visualization, writing – review and editing, software, project administration, data curation. **Seunghyup Yoo**: funding acquisition, writing – review and editing, validation, project administration, supervision, resources, data curation. **Sangin Hahn**: conceptualization, investigation, writing – original draft, methodology, validation, visualization, writing – review and editing, formal analysis, data curation.

## Conflicts of Interest

The authors declare no conflict of interest

## Supporting information




**Supporting File**: advs75894‐sup‐0001‐SuppMat.pdf.

## Data Availability

Research data are not shared.
