# Supplementary material for: Bias‐Tunable Two‐Terminal Organic Photodetector for Intelligent Imaging
Source: Adv Sci (Weinh). 2026 Jun 1;13(48):e75894. doi: 10.1002/advs.75894 (PMC13336377; doi:10.1002/advs.75894)
Supplement: Supplementary file 1 [file ADVS-13-e75894-s001.pdf]

Supporting Information

Bias-Tunable Two-Terminal Organic Photodetector for Intelligent Imaging

Sangin Hahn†, Sanghoon Park† and Seunghyup Yoo*

*Corresponding author: Seunghyup Yoo, syoo.ee@kaist.edu

This file includes:

Figure S1. Equivalent circuit model

Table S1. Fitted parameters via numerical modeling of the *I-V* curve

Figure S2. Determination of the optical bandgap from Tauc plots

Figure S3. Ultraviolet photoemission spectroscopy (UPS) characterization of the deposited films

Table S2: Summary of the energy levels extracted from measurements

Figure S4. Schematic energy band diagram based on the measured parameters

Figure S5: Lifetime comparison of photodetectors with and without encapsulation

Figure S6. TCAD simulation results

Table S3. Simulation parameters used in the TCAD simulation.

Figure S7. Dark current characteristics of the fabricated unit device

Figure S8. Noise spectral density under 1 V operation bias

Figure S9. Optoelectronic characteristics of the planar heterojunction photodiode

Figure S10: Calculated field distribution within the suggested tri-layer device

Figure S11: Responsivity spectrum of the trilayer device

Figure S12: *I-V* curves of the under the blue LED (peak wavelength of 467 nm)

Table S4. Detailed performance comparison with previous works

Figure S13. *I-V* curves of the unit device before and after the cyclic test

Table S5. Representative examples of backplane-integrated thin-film optoelectronic devices

Figure S14. Design layout of 4 × 4 photodetector array

Figure S15. Fabrication process of the photodetector array (top view)

Figure S16. Fabrication process of the photodetector array (cross view)

Figure S17. Response speed of the pixel in fabricated photodetector array

Figure S18. Kernels of simulated neural network: before/after training

Figure S19: Learning curves of simulated neural networks under ideal and nonideal conditions.

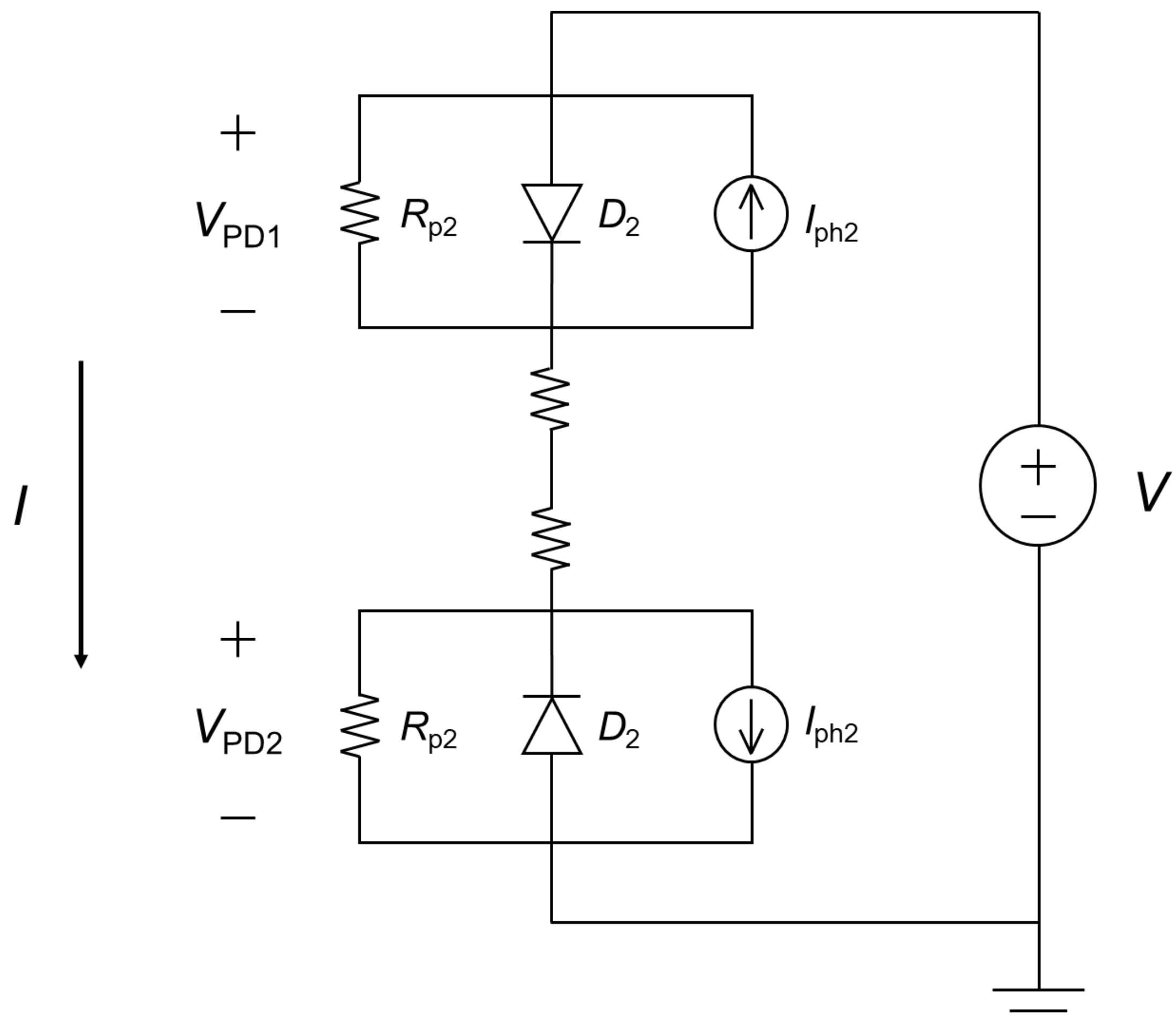

Figure S1. Equivalent circuit model

Parameter	Value	Parameter	Value
J_{s1} (A/cm^2)	1.05×10^{-9}	J_{s2} (A/cm^2)	2.89×10^{-12}
n_1	2	n_2	1.19
$R_{s1}A$ (Ω cm^2)	2.29×10^{3}	$R_{s2}A$ (Ω cm^2)	1.38×10^{3}
$R_{p1}A$ (Ω cm^2)	7.05×10^{4}	$R_{p2}A$ (Ω cm^2)	1.25×10^{5}
J_{ph1} (A/cm^2)	2.72×10^{-5}	J_{ph2} (A/cm^2)	9.19×10^{-5}

Table S1. Fitted parameters via numerical modeling of the *I-V* curve. The active area of the device A is 0.085 cm^2.

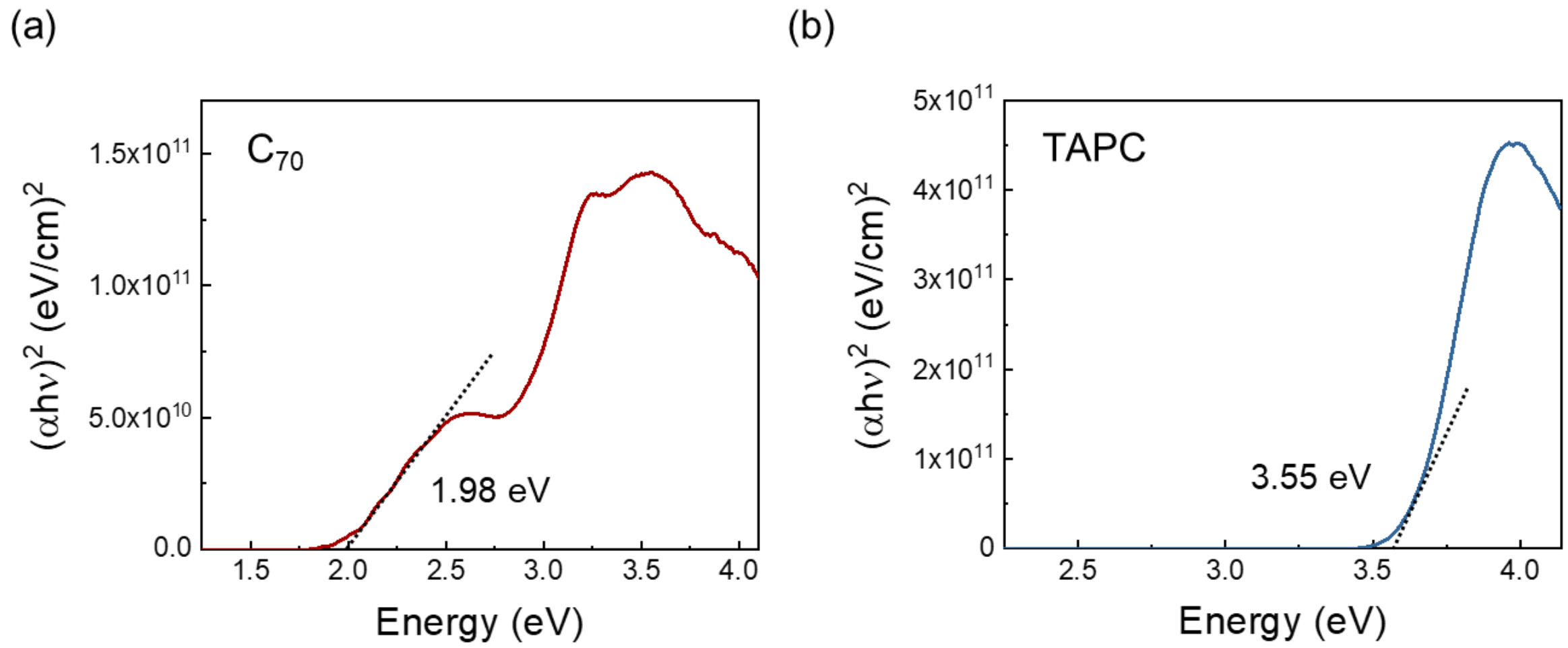

Figure S2. Determination of the optical bandgap of (a) C_{70} and (b) TAPC using Tauc plots derived from UV-Vis absorption spectra.

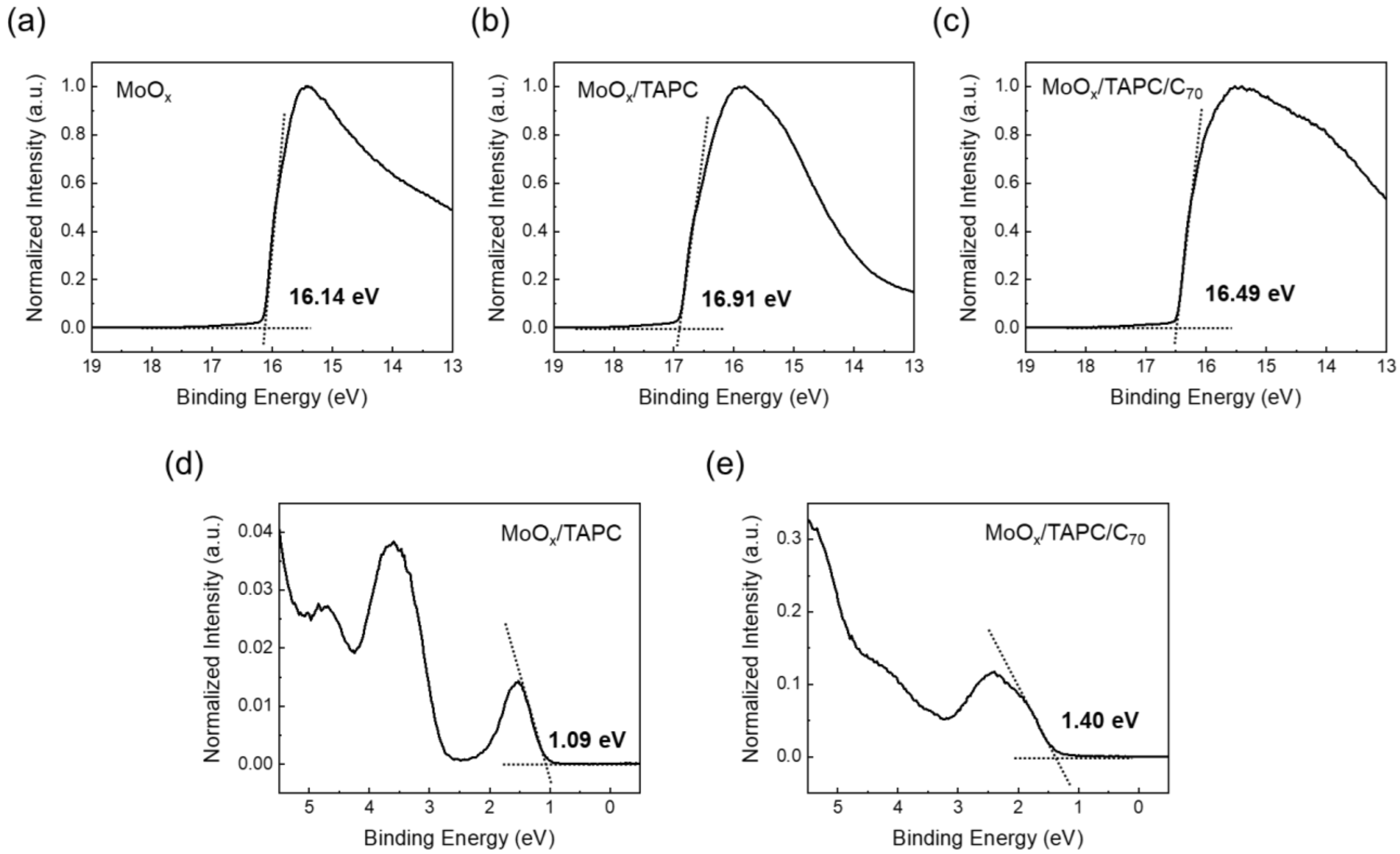

Figure S3. Ultraviolet photoemission spectroscopy (UPS) characterization of the deposited films. The secondary electron cutoff levels (E_{cutoff}) are shown for (a) MoO_x, (b) MoO_x/TAPC, (c) MoO_x/TAPC/C_{70}. The lowest binding energy onset levels (E_{onset}) are shown for (d) MoO_x/TAPC, and (e) MoO_x/TAPC/C_{70}.

The energy levels of the materials including the workfunction (Φ) and the HOMO level (E_{HOMO}) were evaluated using UPS measurements with a He I (hv = 21.22 eV). The Φ was determined from the E_{cutoff} according to the relation $\Phi = hv - E_{cutoff}$. Furthermore, the E_{HOMO} was derived by combining the Φ and the E_{onset} representing the HOMO edge, using the equation $E_{HOMO} = \Phi + E_{onset}$.

Material	Energy Levels	Value
MoO_x	Work function	5.3 eV
TAPC	HOMO	5.40 eV
	LUMO	1.85 eV
	Band gap	3.55 eV
	Work function	4.31 eV
C_{70}	HOMO	6.13 eV
	LUMO	4.15 eV
	Band gap	1.98 eV
	Work function	4.73 eV

Table S2. Summary of the energy levels extracted from UPS measurements and Tauc plots. The WF and HOMO levels were determined from UPS spectra, and the optical bandgaps were estimated using Tauc plots. The LUMO levels were calculated from the measured HOMO levels and optical bandgaps.

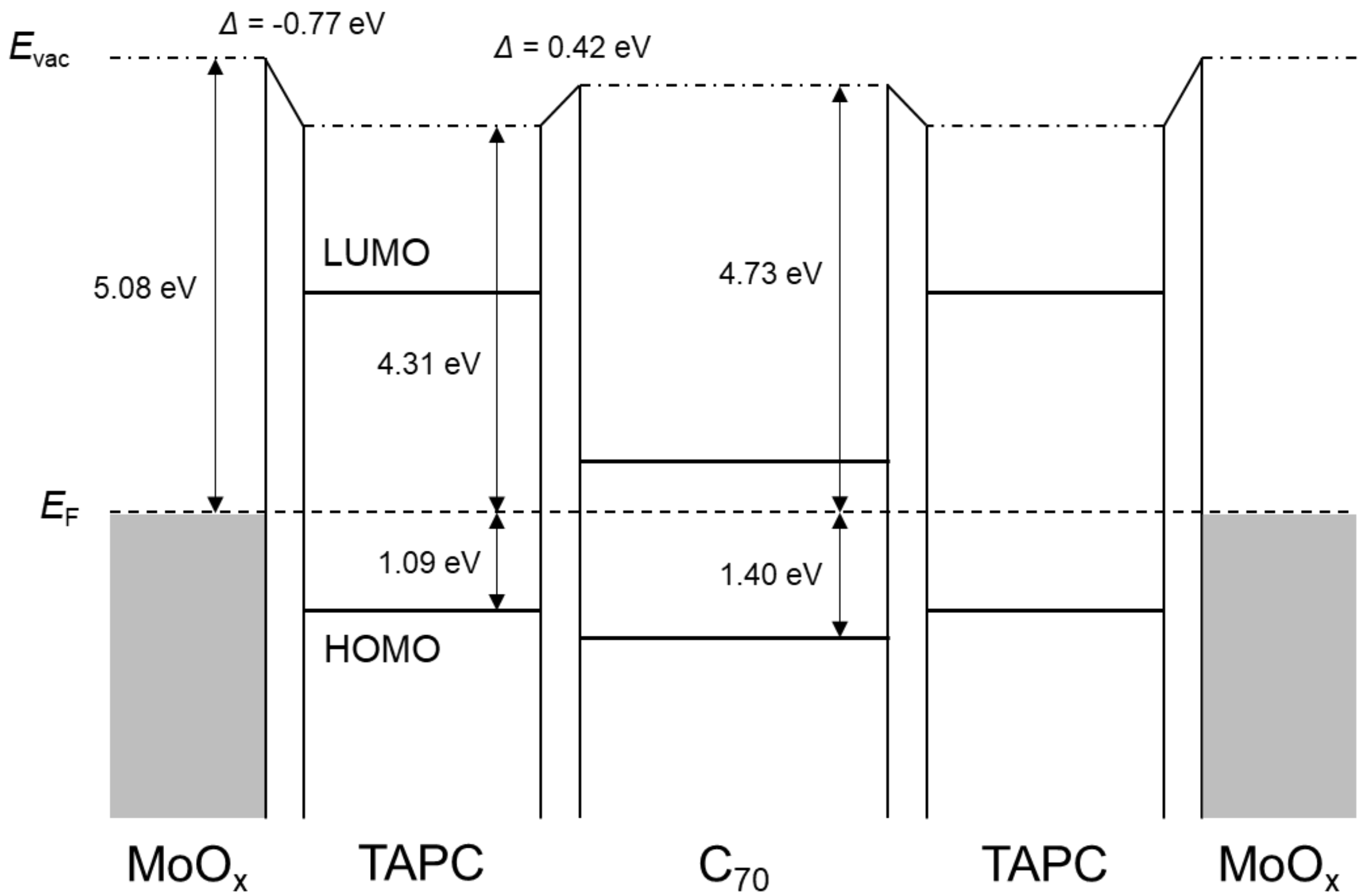

Figure S4. Schematic energy band diagram based on the parameters extracted from UPS and UV-Vis spectra. The energy levels at the interfaces within the device stack are aligned with respect to the work functions, illustrating the vacuum level shifts (*Δ*) caused by interface dipoles.

The energy band diagram is constructed by aligning the energy levels with respect to the measured work functions. Clear vacuum level shifts (Δ) are observed at the interfaces, confirming the formation of interface dipoles. Specifically, the interface dipole at the MoO_x/TAPC interface was determined to be -0.77 eV, while the TAPC/C70 interface exhibited a dipole of 0.42 eV.

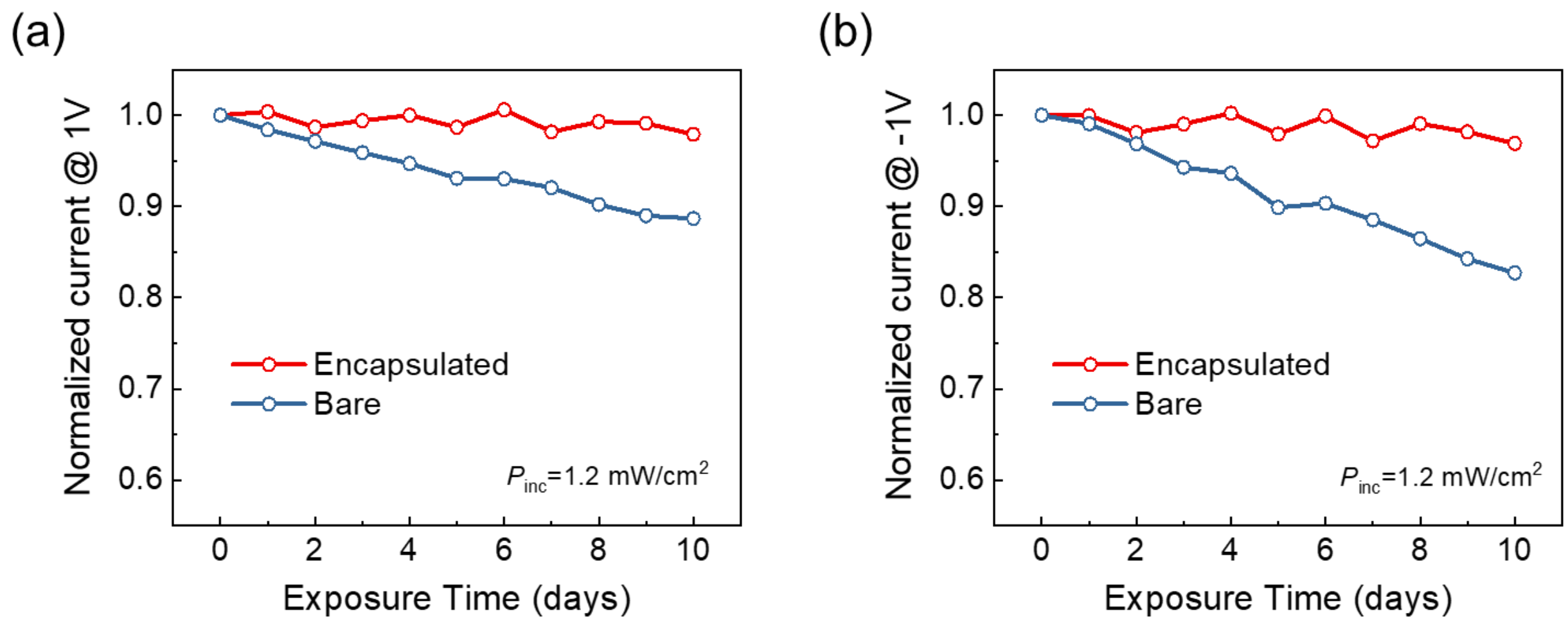

Figure S5: Lifetime comparison of photodetectors with and without encapsulation, by measuring photocurrent (irradiance: 1.2 mW/cm^2) responses under (a) 1 V and (b) -1 V of operation biases. Devices are stored in the ambient air condition.

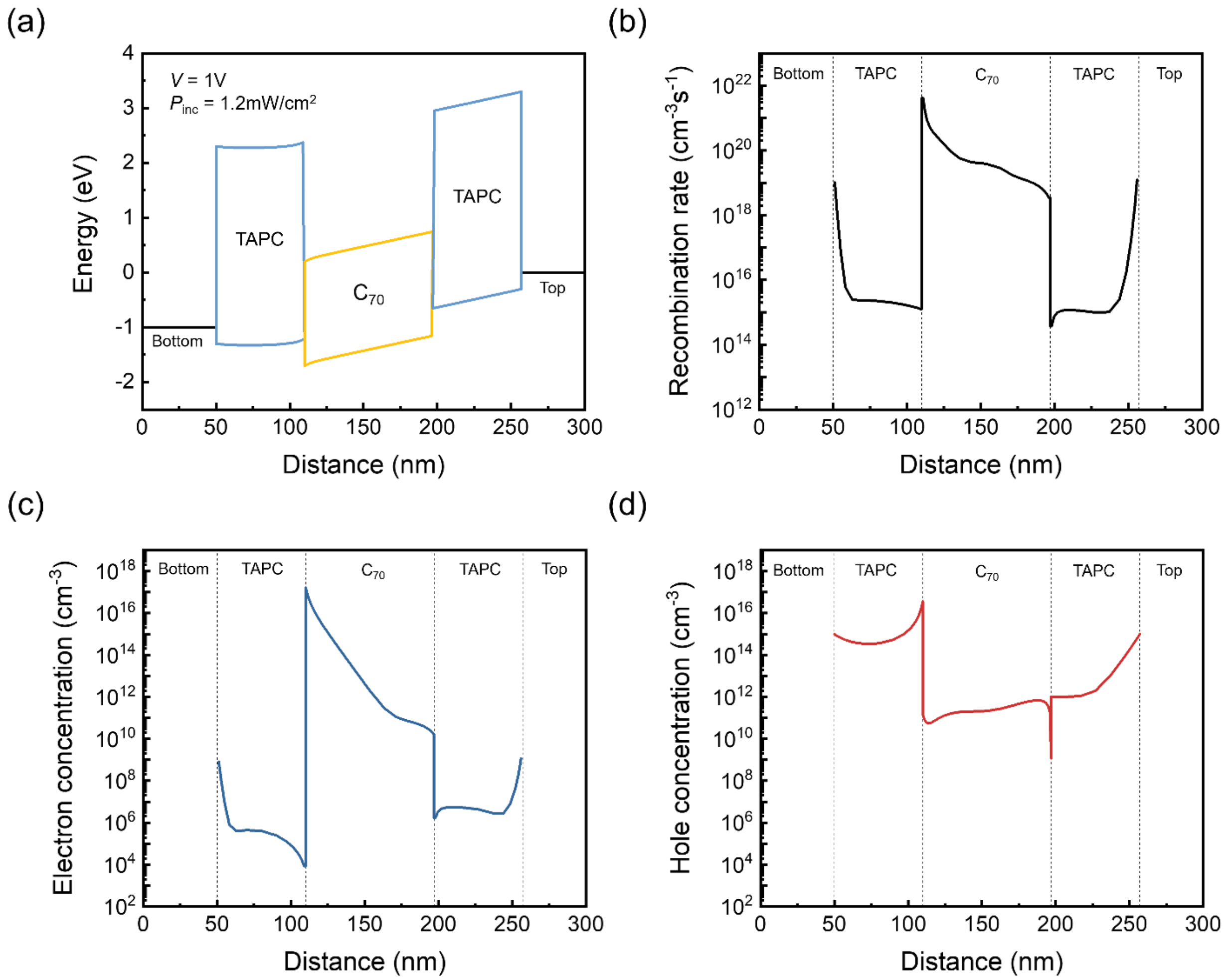

Figure S6. TCAD simulation results including (a) Simulated energy band diagram of the suggested reconfigurable photodetector, (b) Recombination rate, (c) Electron concentration, (d) Hole concentration across the device under illumination.

The high recombination rate at the left-side interface between the TAPC layer and the C_{70} layer indicates that most photogenerated electrons recombine with the holes injected from the bottom electrode.

Material	Paramter	Value
MoO_x	Work function	5.3 eV
TAPC	LUMO	2 eV
	HOMO	5.6 eV
	Band gap	3.6 eV
	Dielectric constant	4
	Electron mobility	10^{-3} $cm^2V^{-1}s^{-1}$
	Hole mobility	10^{-2} $cm^2V^{-1}s^{-1}$
	Conduction band density of states (N_C)	1×10^{20} cm^{-3}
	Valence band density of states (N_V)	1×10^{20} cm^{-3}
C_{70}	LUMO	4.2 eV
	HOMO	6.1 eV
	Band gap	1.9 eV
	Dielectric constant	4
	Electron mobility	0.1 $cm^2V^{-1}s^{-1}$
	Hole mobility	10^{-2} $cm^2V^{-1}s^{-1}$
	Conduction band density of states (N_C)	1×10^{20} cm^{-3}
	Valence band density of states (N_V)	1×10^{20} cm^{-3}

Table S3. Simulation parameters used in the TCAD simulation.

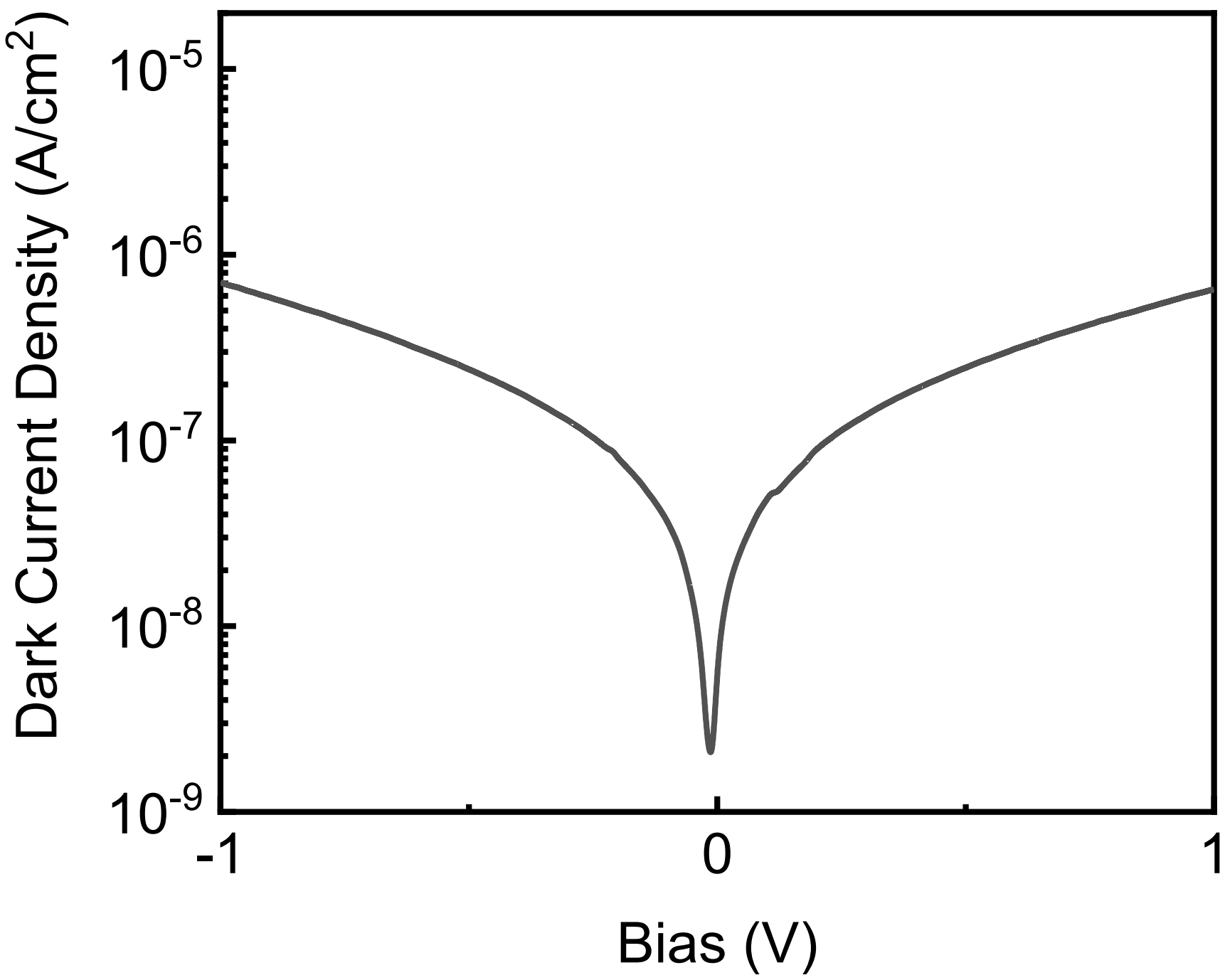

Figure S7. Dark current characteristics of the fabricated unit device

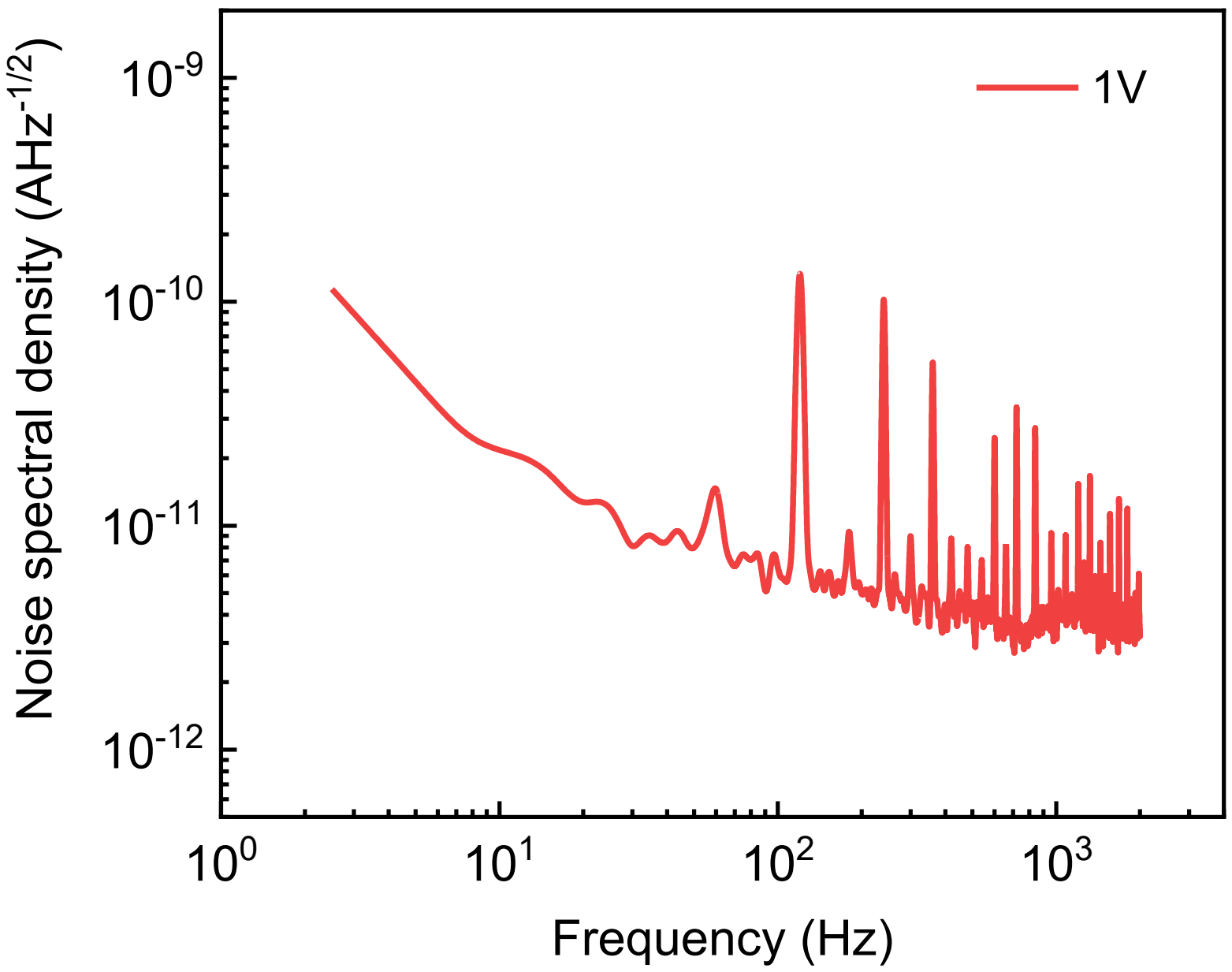

Figure S8. Noise spectral density under 1 V operation bias. The measured noise spectral density was 6.48×10^{-12} $AHz^{-1/2}$ at 100 Hz, therefore calculated spectral detectivity of the device was 3.46×10^{9} Jones. (535 nm wavelength, 1 Hz bandwidth)

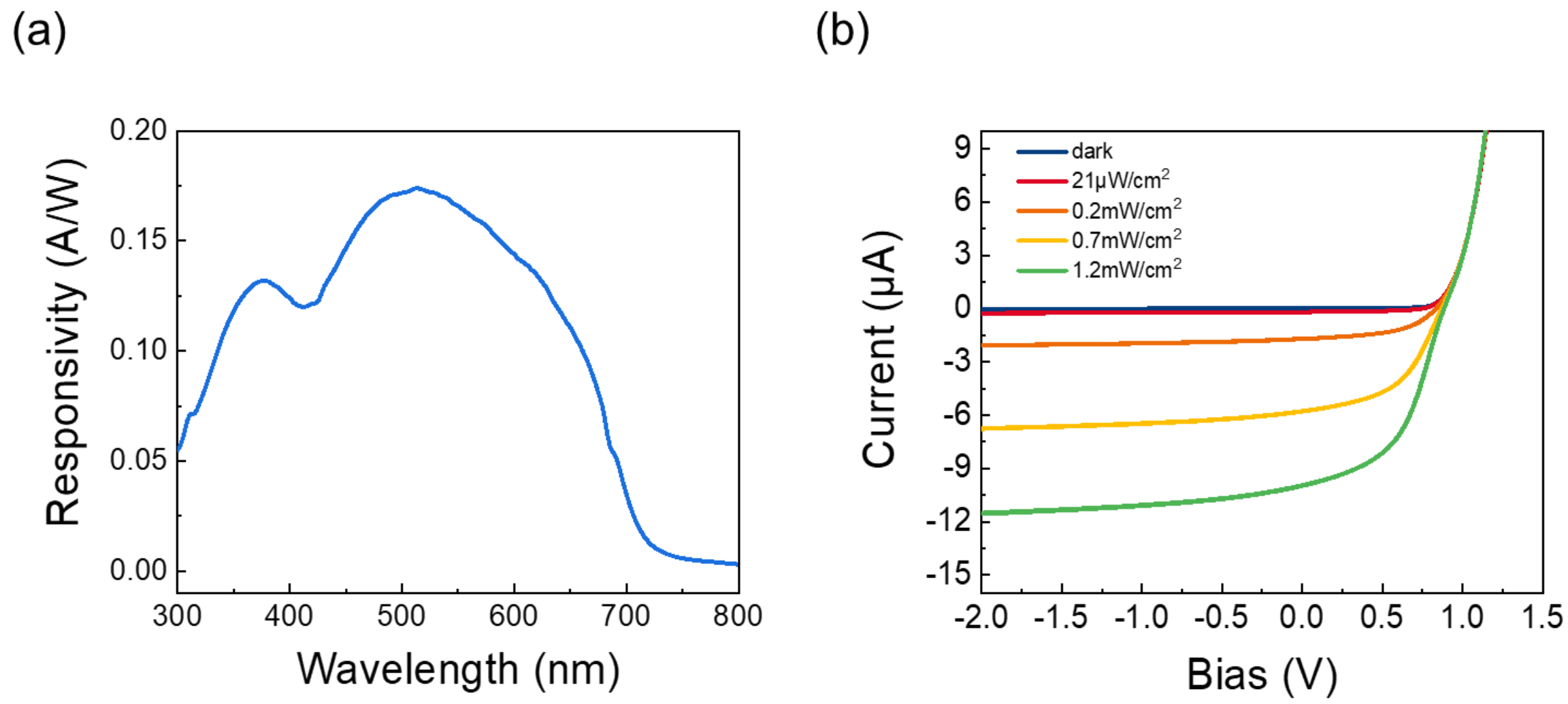

Figure S9. Measured optoelectronic characteristics of planar heterojunction photodiode. (Device structure: ITO 150 nm | MoO_x 5 nm | TAPC 60 nm | C_{70} 87 nm | BPhen 8 nm | Al 100 nm) (a) Responsivity spectrum of the photodiode. (b) *I*-*V* curves of the photodiode under different illumination conditions. (dark to 1.2mW/cm^2, LED with peak wavelength of 535 nm)

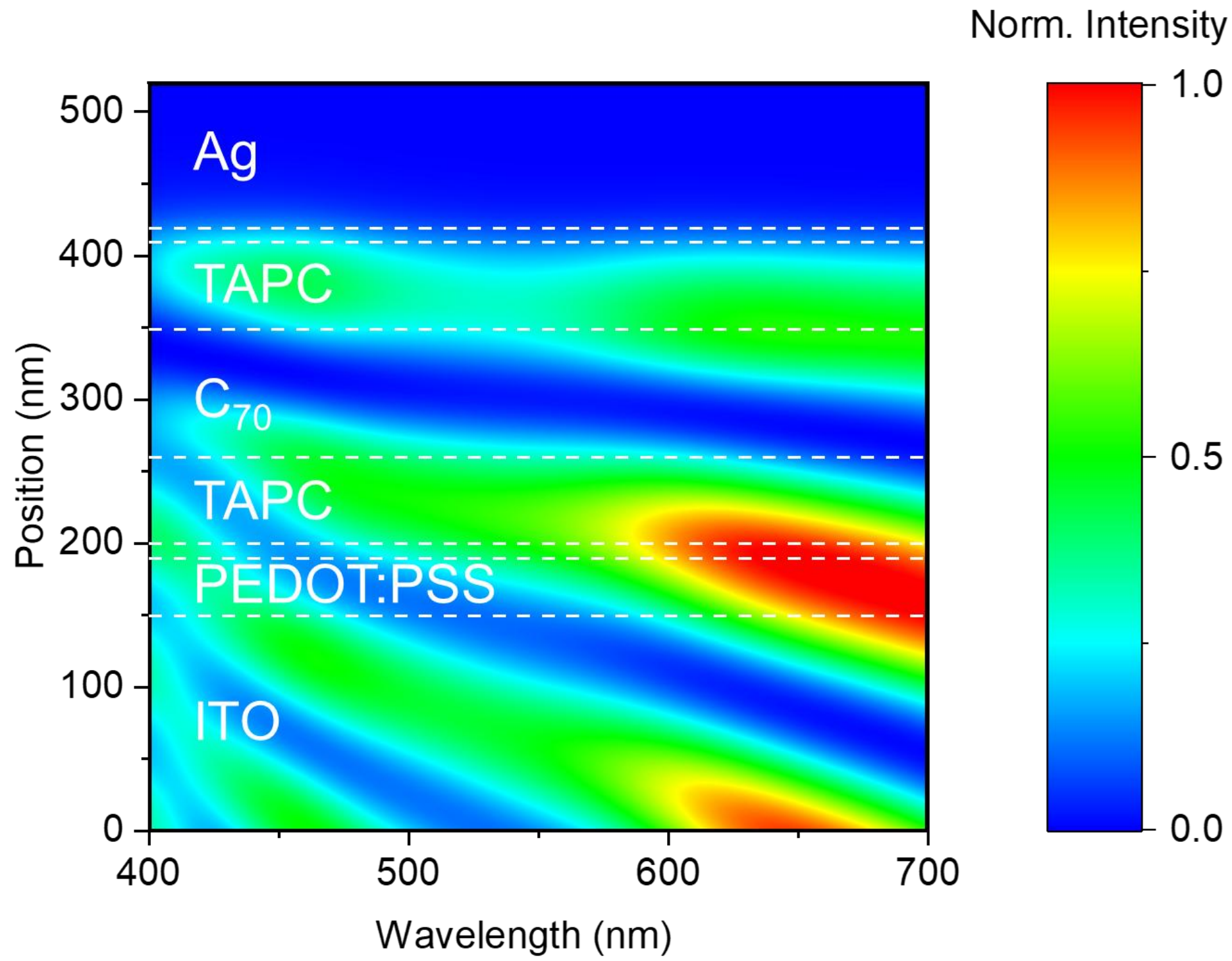

Figure S10: Calculated field distribution within the suggested tri-layer device.

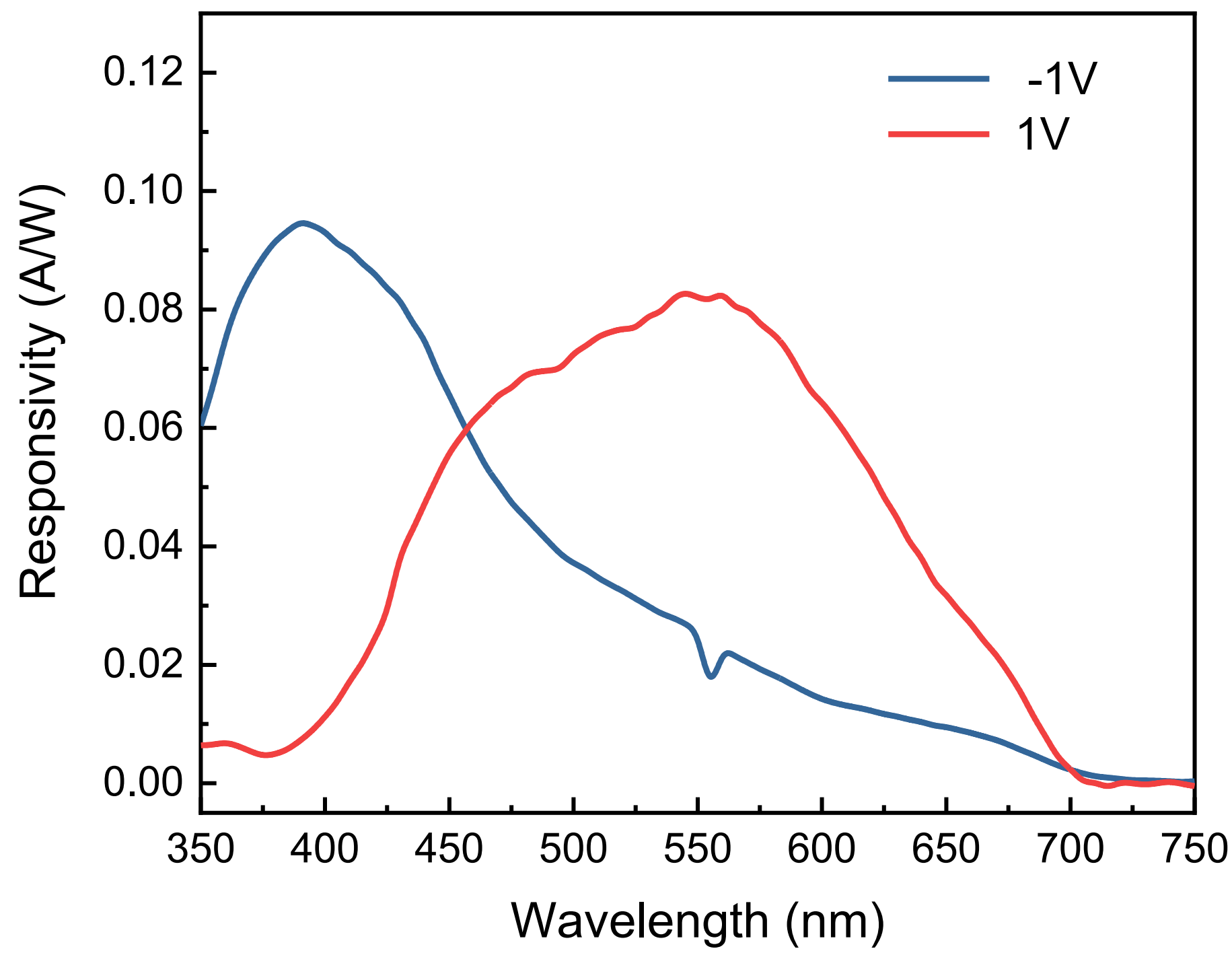

Figure S11: Responsivity spectrum of the trilayer device under positive (1 V, lower junction) and negative (-1 V, upper junction)

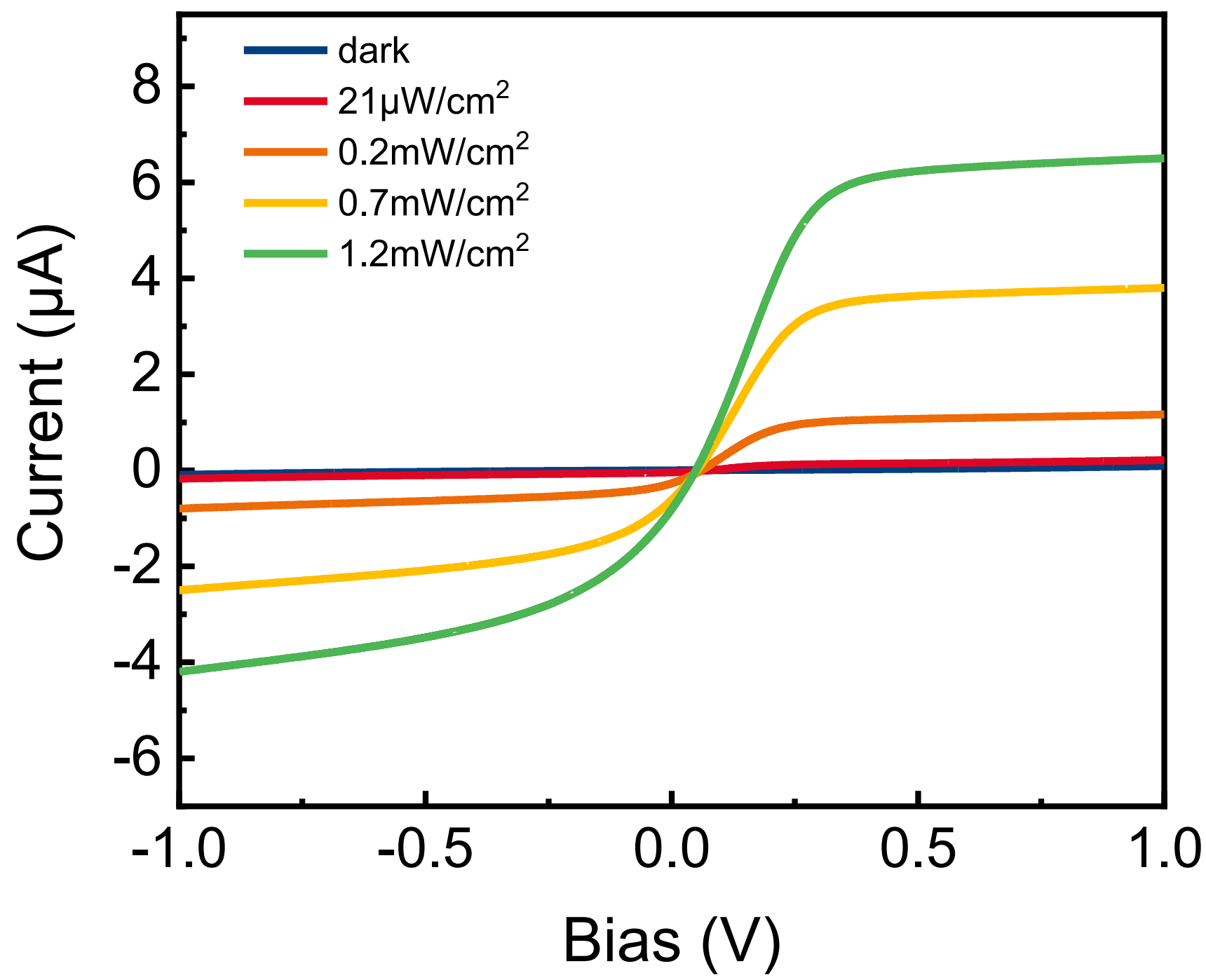

Figure S12: *I-V* curves of the under the blue LED (peak wavelength of 467 nm)

Reference	Configuration (Terminal)	Active Materials	Responsivity Range	Response Time
**This Work**	**D/A/D trilayer (2T)**	**TAPC/C_{70}**	**-26 to 77 mA/W**	**Rise: 0.44 ms Fall: 0.47 ms**
Mondal, S. et al. (12)	Widegap photoconductor (2T)	Ferroelectric ScAlN	R_{max} = 15 mA/W	< 60ms
Jang, H. et al. (26)	Dual gate-controlled FET (4T)	Si	-26 to 26 mA/W (roughly calculated)	Rise: 3 ns Fall: 12 ns
Wang, Y. et al. (27)	Memristor-like (2T)	$CuInP_2S_6$ + Graphene	-16 to 11 mA/W (roughly calculated)	10 ms to 1 s for poling
Oh, G. et al. (21)	Gate-tunable ambipolar FET (3T)	$MoSe_2$ + graphene + ion gel	R_{max} = 285 mA/W	N/A
Wang, Y. et al. (55)	Type-II heterojunction (2T)	$Ge_2Sb_2Te_5/MoS_2$	10.09 mA/W to 1.03 A/W (positive only)	Rise: 150 µs Fall: 288 µs

Table S4. Detailed performance comparison with previously reported representative literature values

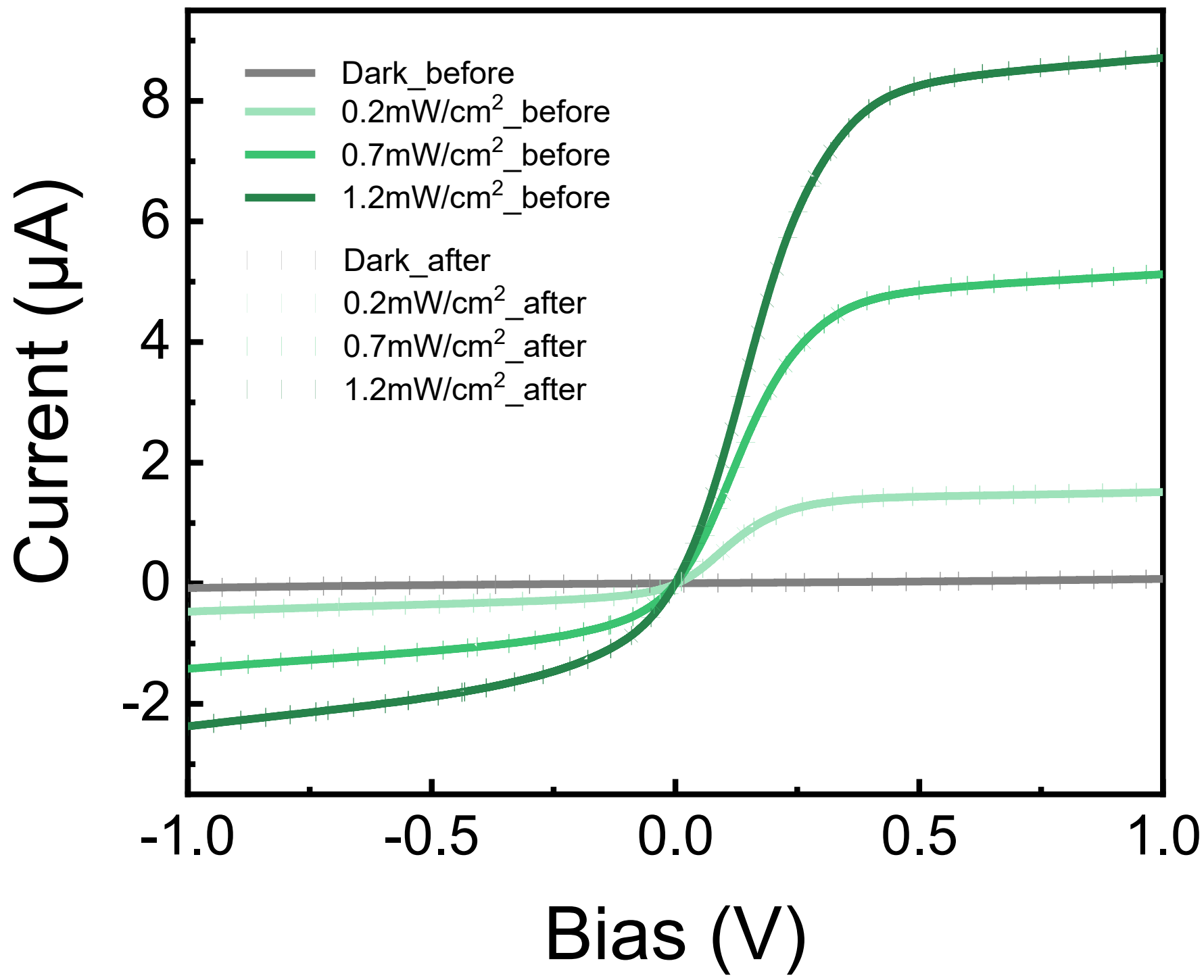

Figure S13. *I*-*V* curves of the unit device before and after the cyclic test

Reference	Device configuration (substrate)	Array resolution	Pixel pitch	Note
J. Lee, et al. (56)	cQD-PD on CMOS ROIC (Si)	64 × 64	5 µm	Thin-film PPD on 4T APS ROIC, $6.1e^-$ of read noise
D. Tordera et al. (57)	Organic PD on 1T TFT backplane (Glass)	640 × 480	50 µm (508 ppi)	High-resolution NIR imager for fingerprint sensing
H. Cho et al. (58)	OLED on CMOS driving circuit (Si)	1280 × 1024	11 µm (2350 ppi)	OLED microdisplay with a tandem structure
S. Heo et al. (59)	OLED on CMOS driving circuit (Si)	N/A	6 µm (4032 ppi)	OLEDoS with complex 7T1C operation circuit
K. Nishimura et al. (60)	Organic PD on 3T APS + feedback (Si)	8192 × 4320	3 µm	High-resolution thin-film imager with a global shutter
J. Lieu et al. (61)	cQD-PD on CMOS ROIC (Si)	640 × 512	15 µm	NIR QD imager with dual conversion gain
H. Kim et al. (62)	RGB QD-microdisplay on ITO bottom (Glass)	10 × 10	6 / 8 µm (4000 ppi)	Direct, sequential patterning of RGB QD active layers
S. Baek et al. (63)	Conventional CMOS image sensor (Si)	N/A	0.45 µm	Current state-of-the-art CIS for reference

Table S5. Representative examples of backplane-integrated thin-film optoelectronic devices, including thin-film photodetector-based imaging platforms and vertically stacked devices on switching backplanes.

The listed works show that micron-pitch pixel miniaturization is determined mainly by backplane configuration, thereby highlighting thin-film devices including the present work can offer a direct route toward high-resolution array implementation with their robust integration capability. Previous reports support the validity of this approach for implementing high-resolution pixel array with few microns of pixel pitch, since the level of carrier mobility of each component with nanometer-scale film thickness allows vertical photocarrier transport while limiting lateral leakage paths.

However, further pixel miniaturization toward sub-micron levels like recently reported state-of-the-art CIS introduces several practical challenges. First, as the pixel pitch decreases, lower noise floor is required since the amount of harvested light follows the size of the active area. In particular, lateral crosstalk between adjacent pixels becomes increasingly important to integrate thin-film devices on backplanes since normally integration process does not include pixel wise patterning of each interlayer. This configuration could introduce weak parasitic carrier pathways thereby degrading image fidelity and reduce effective modulation contrast. Moreover, the fill factor can no longer be assumed to remain high during the miniaturization since the interconnect/layout constraints begin to dominate effective active aperture.

To address these issues, several strategies are being actively explored in both field of micro-display and image sensors. One main approach is direct pixel-wise photopatterning of thin-film device stack to localize the active regions, with optimized backplane co-design to match and operate the device stack above. In this context, the main advantage of the present platform in a view of array

implementation is that the active stack itself does not require direct microscale patterning, which leaves room for scalable integration; however, realizing ultrahigh-density image sensors will require coordinated optimization of the backplane, pixel-definition scheme, and thin-film patterning process to preserve fill factor and maintain the image quality.

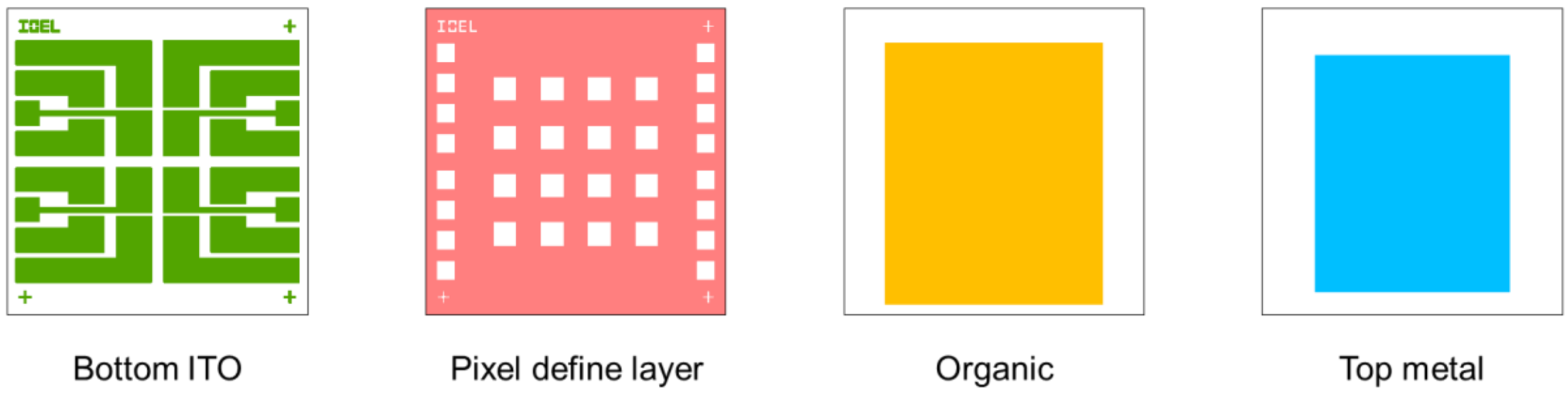

Figure S14. Design layout of 4×4 photodetector array

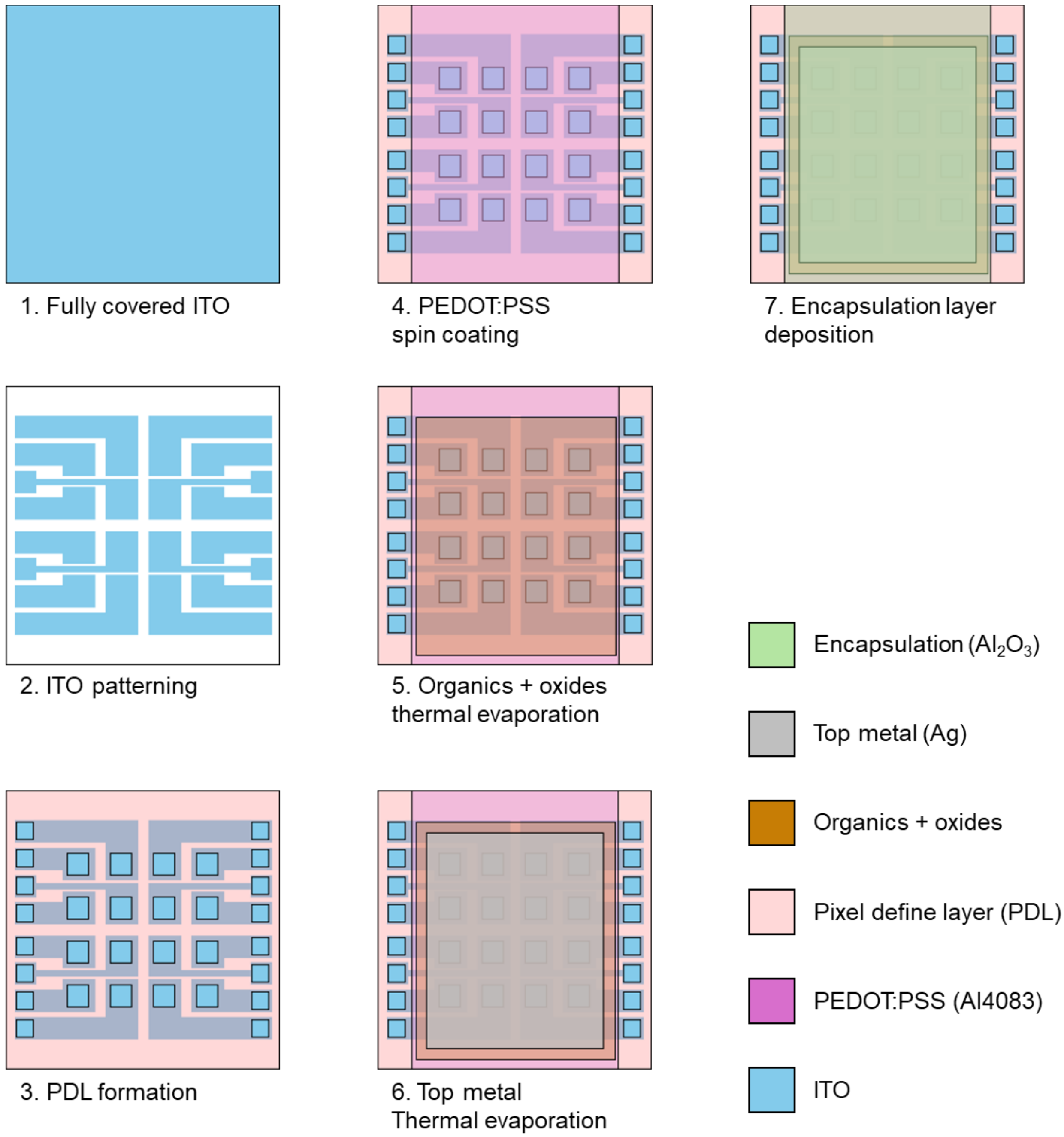

Figure S15. Fabrication process of the photodetector array (top view)

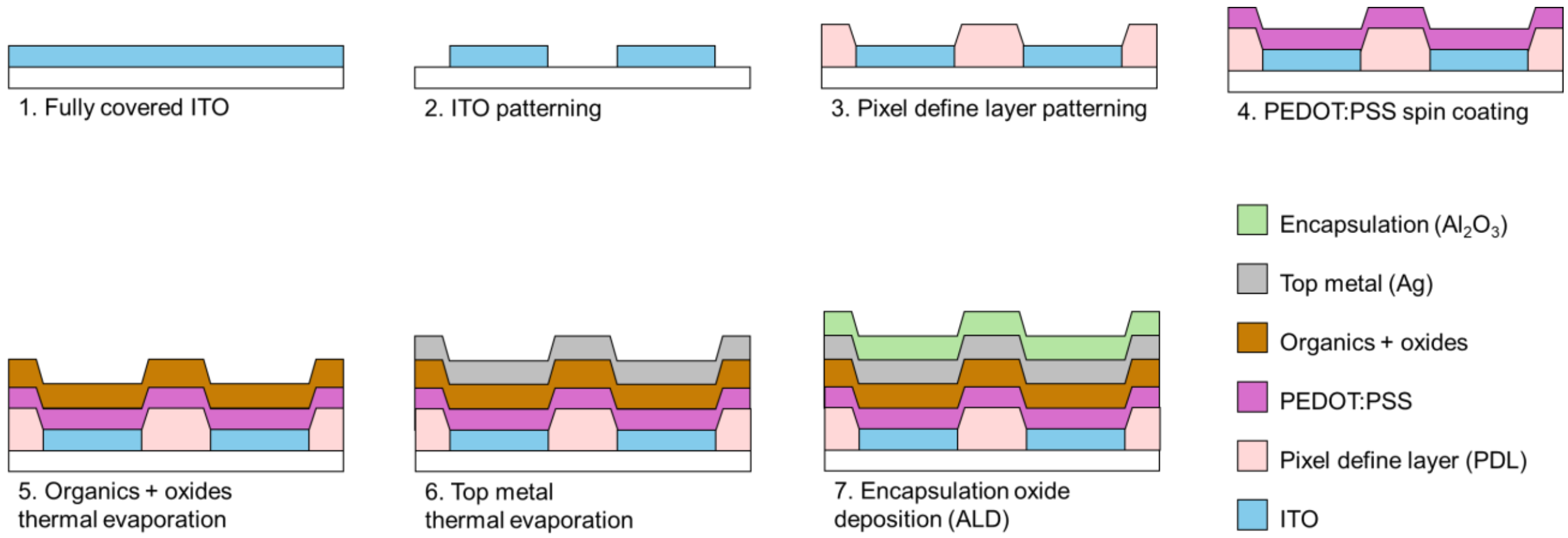

Figure S16. Fabrication process of the photodetector array (cross view)

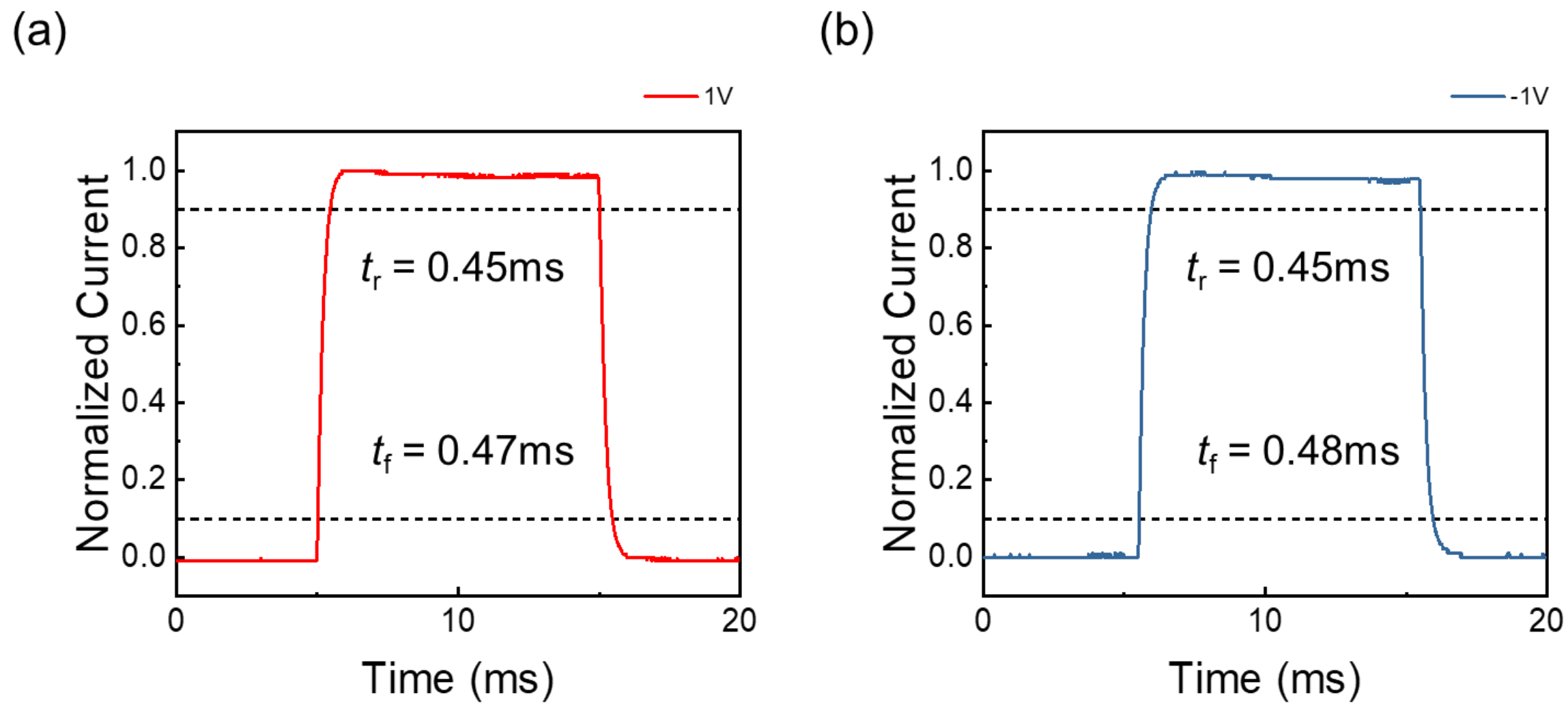

Figure S17. Response speed of the pixel in fabricated photodetector array, with operation bias conditions of (a) 1 V and (b) -1 V. Measured rise times under 1 V and -1 V were both 0.45 ms, while fall times were 0.47 ms and 0.48 ms

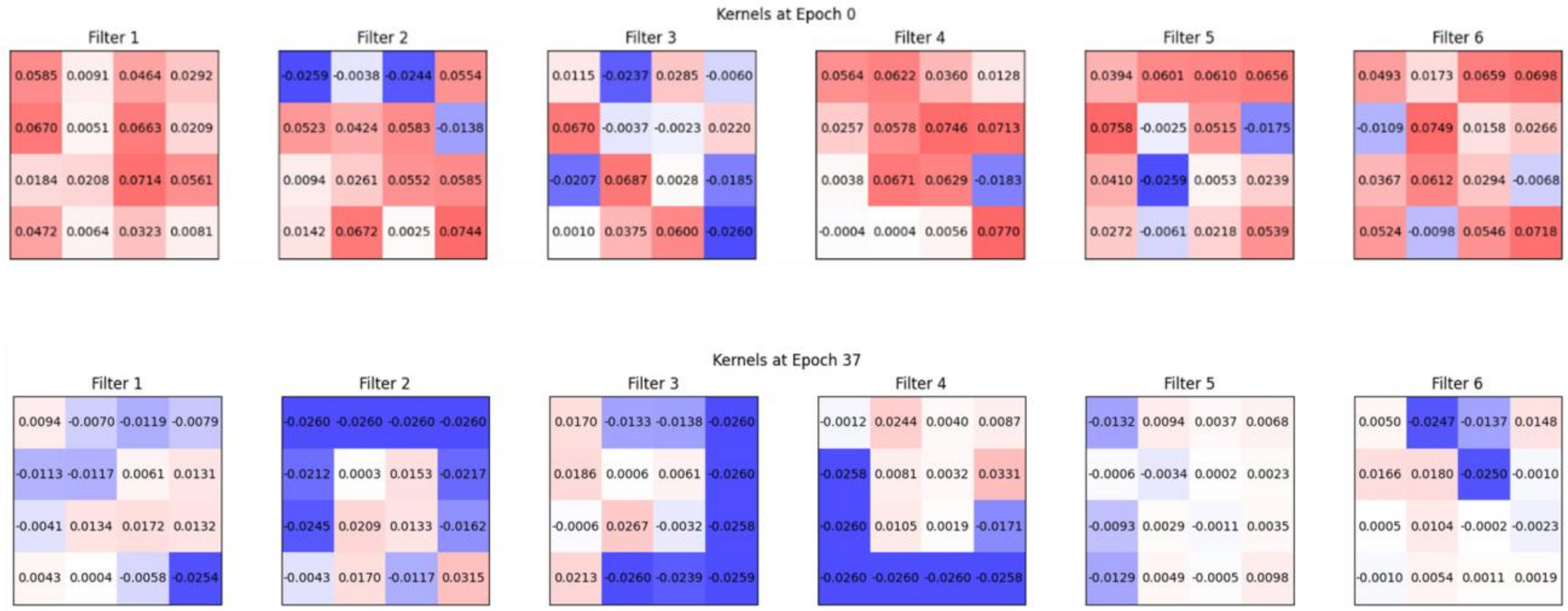

Figure S18. Kernels of simulated neural network: before/after training

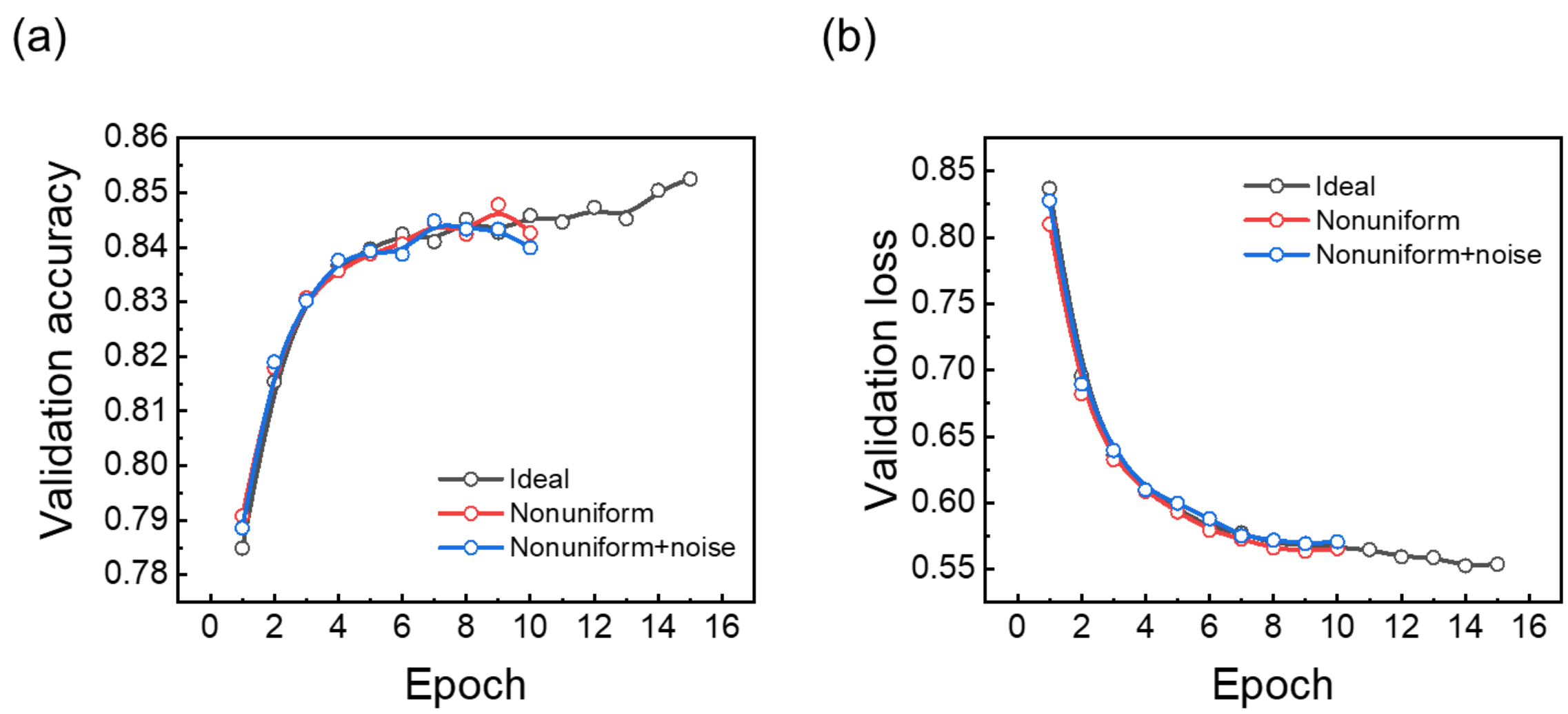

Figure S19: Learning curves of simulated neural networks under ideal and nonideal conditions. From (a) validation accuracy and (b) validation loss curves, we confirm that despite the introduction of these non-ideal factors, the simulated learning curves showed no significant degradation, maintaining a stable convergence behavior comparable to the ideal case
